# Life Course Social Mobility and Parenthood. Counterfactual Estimates of the Motherhood Class Penalty in Britain

**DOI:** 10.1111/1468-4446.70039

**Published:** 2025-10-21

**Authors:** Giacomo Vagni

**Affiliations:** ^1^ University of Essex Colchester UK

**Keywords:** causality, motherhood, penalty, sequence analysis, stratification

## Abstract

This paper investigates the causal effect of motherhood on women's occupational class trajectories—the Motherhood Class Penalty—using data from the 1970 British Cohort Study. We apply sequence optimal matching alongside other matching techniques to construct counterfactual class trajectories for mothers in the UK. Our results show that motherhood significantly increases downward mobility and limits access to professional occupations. Low professional women face an estimated 15% penalty, while high professional women experience a 5% penalty compared to their childless counterparts. We find that professional‐class women are more likely to remain attached to the labour market after childbirth, whereas working‐class mothers are at greater risk of permanently exiting the workforce. Among all groups, low professional women experience the most significant forgone upward mobility, highlighting how motherhood penalties vary across the class spectrum. These findings stress the substantial human capital loss associated with motherhood in the UK and suggest that occupational penalties are shaped by existing socio‐economic hierarchies, potentially reinforcing broader patterns of inequality.

## 1 Introduction

The transition to parenthood has far‐reaching consequences for women's careers. Even among those in high‐status occupations, many experience a decline in wages; this effect is known as the Motherhood Penalty (Waldfogel 1997). While a substantial body of research has examined the economic costs of motherhood, such as reductions in wages (Budig and England 2001), individual earnings (Kleven et al. 2019), and household income (Musick et al. 2020), its impact on social class mobility has received less attention. This paper seeks to fill that gap by estimating the effect of motherhood on women's class trajectories in Britain.

Analysing the motherhood penalty through the lens of occupational class offers a rich understanding of the structural barriers women face in the labour market. Career advancement is rarely linear and is often shaped by class‐based boundaries and mechanisms of social closure (Erikson and Goldthorpe 1992; Weeden and Grusky 2005). Social class captures dimensions of work not fully reflected in earnings—such as long‐term security, opportunities for progression, autonomy, and the transmission of status and capital (Bukodi and Goldthorpe 2018; Rose et al. 2005; Wright 2005). A class‐based perspective, therefore, provides a deeper sociological framework for understanding women's career patterns, which are central to both the motherhood penalty and the gender pay gap (Goldin 2006, 2014).

This paper employs a novel methodology that combines sequence analysis with causal inference to estimate counterfactual occupational class trajectories and quantify what we call the Motherhood Class Penalty (MCP) for British women born in 1970. The MCP represents the causal effect of motherhood on a woman's occupational mobility, which we conceptualise as her entire occupational class trajectory from age 16 to 42. The MCP is calculated as the difference between a mother's observed trajectory following childbirth and the counterfactual trajectory she likely would have experienced during the same period had she remained childless.

To achieve this, we develop and apply a novel method we call Individual Synthetic Sequence Matching (ISSM). As a methodological contribution, ISSM represents an advance for estimating causal effects with longitudinal categorical data such as occupational histories. The method is highly flexible, enabling researchers to formally incorporate rich pre‐treatment sequence information into the matching process to analyse the complex dynamics of life course trajectories.

Our analysis shows that motherhood substantially reduces upward women's occupational mobility, particularly their likelihood of attaining or remaining in professional and managerial roles. Specifically, we find that motherhood decreases the probability of occupying lower professional positions by around 15 percentage points, and higher professional positions by 5 percentage points. This 20‐point shortfall is largely driven by mothers exiting the labour market into family care, women who, counterfactually, may have sustained or achieved professional status.

The UK presents a compelling case study because its political economy is situated between the liberal welfare regime of the United States and the more 'decommodified' models of continental Europe (Esping‐Andersen 1990; Schmidt 2002). While the UK labour market is more regulated than that of the US, it remains far more liberalised than its European counterparts. The same applies to family policies: although some protections for working mothers exist, unlike at the federal level in the US, they are considerably more limited than those found across the rest of Europe. These features make the UK a key intermediate case for understanding how a hybrid market model shapes the occupational penalties of motherhood.

The paper is structured as follows. We first review the literature on motherhood, employment, and social mobility, then present our data and analytical strategy. We then detail the results on class mobility and penalty estimates, and conclude with a discussion of the findings, their implications, and limitations.

## 2 Background

### 2.1 Women's Occupational Mobility in Britain

Recent studies on social mobility show an important increase in both absolute and relative mobility for women born in the 1970s compared to those born immediately after World War II (Bukodi et al. 2017). In the latter half of the 20^th^ century, each new cohort of women showed higher levels of social mobility than previous generations. This upward trend is attributed to a combination of factors, including anti‐discrimination laws, parental leave policies, the rise of female‐dominated white‐collar occupations, and increased access to education (Bryson et al. 2020; Bukodi et al. 2016; Dex et al. 2008). Women's representation in high‐status occupations has also increased, from just 4%–5.3% in 1980/81% to 11.1% in the 2000s (Dex et al. 2008).

Despite these advances, significant gender disparities remain, particularly in the managerial class, where women are still underrepresented. For example, the 2021 UK Census revealed that men held 7% more higher managerial roles than women (Office for National Statistics 2023). Additionally, women are more likely than men to begin their careers in lower‐level occupations (48% of women vs. 30% of men in the 1970 cohort) and are less likely to experience upward mobility (Bukodi et al. 2017).

The changes in women's occupational structure and mobility are complex because the trajectories of women in full‐time occupations and those in part‐time occupations are different, and women's career trajectories are more affected by life course events, such as parenthood, than those of men. In other words, there is greater heterogeneity in women's occupational trajectories, and this heterogeneity is likely to be socially stratified (Office for National Statistics 2023). For instance, women in working‐class occupations are much more likely to have spells of part‐time employment (Dex and Bukodi 2012). This is consequential because spells of part‐time employment, due to, for instance, the transition to parenthood, hinder career growth and, ultimately, occupational mobility (Costa Dias et al. 2020; Dex and Bukodi 2012). Therefore, the social fluidity of women (relative social mobility) of full‐time versus part‐time women is different and appears to have different generating mechanisms (Bukodi et al. 2017). It is not surprising that part‐time status is associated with lower‐status occupations, non‐standard working schedules, lower pay, and is generally more precarious (Leoncini et al. 2024). For reference, in 2024–2025, it is estimated that about 36% of women work part‐time compared to 15% of male employees (Francis‐Devine et al. 2025).

### 2.2 Motherhood Penalty in Pay and Occupations

#### 2.2.1 Wages and Earnings

The ‘Motherhood Penalty’ refers to the negative impact that having children has on women's wages and earnings, a phenomenon well‐documented in sociological and economic literature (Budig and England 2001). The birth of a first child is often the key factor leading to a long‐term decline in women's wage trajectories (Anderson et al. 2002; Budig and England 2001; Waldfogel 1997). Studies estimate that the wage penalty for women in Britain ranges from 7% to 15%, depending on their employment status (Davies and Pierre 2005; de Linde Leonard and Stanley 2020; Gangl and Ziefle 2009; Kalabikhina et al. 2024).

Overall, earning penalties are higher when accounting for the non‐working women in the population. A comparative study by Kleven et al. (2019) shows a penalty of around 20% in Denmark, 44% in Britain, and 61% in Germany. In contrast, the birth of a child typically has little to no effect on fathers' earnings (Andrew et al. 2021; Icardi et al. 2022; Mari 2019).

#### 2.2.2 Occupational Class Penalty

Dex first investigated the occupational penalty of motherhood in her monograph *Women's Occupational Mobility (1987)*. She noted that ‘women with children have had more experiences of downward mobility than childless women’ (Dex 1987, 71). She also points out that returning to employment after birth in a part‐time position increases the probability of downward mobility (Dex 1987, 83). A series of research on this topic generally confirmed the association between motherhood and occupational mobility patterns (Abendroth et al. 2014; Dex et al. 2008; Jacobs 1999).

Abendroth et al. (2014), using a comparative framework of 13 countries, show an average negative effect of first and second births on occupational status. They find evidence of cumulative penalties on occupational status persisting years after the first birth. Similarly, Aisenbrey et al. (2009) show that short work interruptions in liberal welfare regimes increase the likelihood of downward mobility.

Specific to the UK context, Harkness et al. (2019) report that 77% of mothers return to jobs with the same occupational status after childbirth. Over 5 years, 13% of mothers achieved upward mobility, while 18% of mothers experienced downward mobility. Joshi and Hinde, studying the 1946 cohort, present some descriptive statistics on occupational downgrading after giving birth (1993, p. 222). They found that 36% of mothers re‐entered a lower occupation (downward mobility), and 12% re‐entered a job at a higher level (upward mobility). Dex et al. (2008) further explored cohort differences, finding that mothers born in the 1958 cohort had a 28.5% likelihood of downward mobility, compared to approximately 34% for those born in earlier cohorts. Comparing the first to the most recent job for women with and without children, they offer the following findings. Regarding occupational immobility (no change in social class over time), they find that non‐mothers are 11–13 percentage points more likely to remain in the same occupational class compared to mothers. In terms of downward mobility, the most recent cohorts in their study (the 1956 cohort) indicate that mothers are 12 percentage points more likely than non‐mothers to transition into lower‐class occupations compared to their first job.

#### 2.2.3 Motherhood Penalty Heterogeneity

The impact of the motherhood penalty varies significantly across social classes and income levels. Joshi and Hinde (1993) observed that women in professional occupations were more likely to return to the same occupational class after motherhood, with only 20% facing downward mobility. In contrast, mothers in lower occupational classes had only a 50% chance of staying in the same group. For instance, women in office work had a 42% chance of remaining in the same group, a 45% chance of downward mobility, and little chance of upward mobility.

However, this dynamic becomes more complex when considering whether mothers return to full‐time or part‐time work. Dex and Bukodi (2012) found that women in high‐status jobs were more likely (70%) to experience downward mobility if they shifted to part‐time work after childbirth. This is also consistent with the findings from Abendroth et al. (2014) that reductions in work hours and career interruptions around the time of birth account for the immediate decline in occupational status.

The penalty's distribution across income levels is mixed. Some studies find that women at the bottom of the income distribution face higher penalties (Cukrowska‐Torzewska and Matysiak 2020; Vagni and Breen 2021), while others suggest that the penalty is more severe for higher earners. England et al. (2016) found that highly skilled, high‐wage white women experience the largest motherhood penalties (10% per child), a greater penalty than low‐skill, low‐wage women, whose penalties were closer to 5%–7%. This disparity is attributed to the steep wage growth trajectories of highly skilled women, making even brief interruptions particularly costly (Albrecht et al. 2003; Costa Dias et al. 2020; Wilde et al. 2010).

### 2.3 Theories and Empirical Hypotheses

The motherhood penalty can be explained by both supply‐side and demand‐side theories. Demand‐side theories focus on employer biases and institutional structures that perpetuate the penalty. Employer discrimination arises from stereotypes that mothers are less committed or productive workers, leading to fewer promotions and exclusion from high‐status roles (Gough and Noonan 2013; Ishizuka 2021). Statistical discrimination exacerbates these biases, as employers may generalise perceived inefficiencies of motherhood to all women of childbearing age (Correll et al. 2007; Ishizuka 2021). Institutional discrimination further entrenches penalties through workplace norms that favour continuous trajectories (Bukodi and Dex 2010; Costa Dias et al. 2020). Mothers who take breaks or work part‐time face structural disadvantages in promotions and seniority. The broader policy, institutional and cultural context can moderate these effects (Abendroth et al. 2014; Aisenbrey et al. 2009; Harkness and Waldfogel 2003; Kalabikhina et al. 2024).

Supply‐side theories emphasise how mothers' characteristics, behaviours, as well as constraints, contribute to occupational penalties. For instance, Becker's human capital theory (1985) argues that career interruptions for childcare reduce work experience and skill accumulation, leading to occupational downgrading and wage stagnation. Mothers who take extended leave or transition to part‐time work face depreciation in job‐specific skills, limiting their ability to compete for higher‐status roles (Bukodi and Dex 2010; Goldin 2021). The self‐section of mothers into certain occupations plays an important role in explaining the penalty (Wilde et al. 2010). Mothers often self‐select into jobs with flexible hours or reduced demands to accommodate unequal caregiving responsibilities (Altintas and Sullivan 2017; Davies and Pierre 2005; Gough and Noonan 2013). These adjustments frequently entail trade‐offs, such as lower wages or diminished occupational prestige, reinforcing long‐term career penalties.

Based on the literature and theoretical considerations, we make a first general hypothesis:

Hypothesis 1 *Women who become mothers will be more likely to experience downward occupational mobility, are less likely to remain in the same occupational class, and are less likely to experience upward mobility over the course of their careers. Motherhood is thus expected to limit opportunities for upward movement across the occupational hierarchy, and to reinforce downward movement.*

This leads to the following hypothesis regarding which women are most heavily penalised by motherhood. The motherhood penalty is not uniform but is instead deeply stratified by occupational class, reflecting the unequal distribution of job security, contractual protections, and career trajectories available to women in different social positions. Professional and managerial occupations—typically characterised by salaried contracts, benefits, and formal career ladders—offer greater structural protections against the immediate financial shocks of maternity leave. However, these same occupations often impose severe long‐term penalties for career interruptions, as their advancement systems are built on assumptions of continuous, full‐time commitment. In fields such as law, finance, and corporate management, the expectation of uninterrupted productivity means that even brief career breaks can derail promotion timelines, pushing mothers onto slower, often dead‐end tracks (Goldin 2021).

By contrast, working‐class occupations—frequently structured around hourly wages, temporary contracts, and minimal benefits—offer little protection against the economic consequences of motherhood. While these jobs may not penalise intermittent employment in the same way (since turnover is already high), they provide almost no safeguards for pregnant workers or new mothers. Precarious scheduling practices that make balancing childcare very difficult, often forcing mothers into involuntary part‐time work or job loss. The lack of paid leave, unpredictable hours, and employer retaliation for pregnancy‐related absences mean that working‐class mothers face immediate economic instability rather than gradual career stagnation.

Based on these considerations, we formulate the following hypotheses:

Hypothesis 2 *Women in professional‐class occupations prior to motherhood will incur larger long‐term occupational penalties, specifically driven by greater forgone upward mobility, compared specifically to women in working‐class occupations. Because their counterfactual career paths often involve steeper advancement, interruptions due to motherhood lead to larger cumulative deficits in occupational attainment over the long term.*

Hypothesis 3 *Women in working‐class occupations prior to motherhood will experience greater short‐term occupational instability following childbirth, marked by a higher probability of exiting the labour market entirely, compared to women in professional‐class occupations. Because they face more precarious employment conditions with fewer supports, they are more vulnerable to job loss or disruption, making complete withdrawal from employment a more likely outcome of the instability induced by the transition to motherhood.*

## 3 Data and Methods

### 3.1 Data

We use the British Cohort Study 1970 (BCS70) to estimate the MCP in Britain. The data is curated by the UCL Centre for Longitudinal Studies (UCL Social Research Institute 2025).

We focus on occupational social class from age 16 to age 42, which corresponds to Sweep 9 of the BCS70. However, we use Sweep 10, which corresponds to age 46, to check if a woman ever had a child. Unfortunately, we were not able to construct a comparable social class variable for age 46 (see Supporting Information S1: Appendix A). But by the age of 42, most women will have attained a stable class position. We used both the Activity and Partnership datasets (University College London et al. 2023) and the BCS Sweeps.

The analytical sample was derived starting with 18,037 individuals from the British Cohort Study 1970 (BCS70) activity and partnership datasets. The sample was first restricted to the 9811 participants who completed Sweep 9 (age 42), excluding 8226 individuals. After removing 1226 individuals with missing or incomplete activity histories, the sample was reduced to 8585. Following the merge with the main BCS dataset, 1018 individuals were lost due to missing data across key variables, resulting in 5726 complete cases. The sample was then restricted to women only, excluding 2645 men, yielding 3081 women. An additional 146 women who had their first birth before age 20 were excluded to ensure adequate pre‐treatment observation periods for controlling for pre‐birth employment trajectories, resulting in 2935 women. Finally, to create a balanced panel with annual employment observations from age 16 to 42 (26 time periods), 394 women with incomplete observation periods were excluded, resulting in the final analytical sample of 2541 women. This approach of restricting the analysis to a balanced panel with complete observation histories was chosen to avoid the complexities of imputing missing person‐year spells in the trajectory data. Of these, 2017 (79%) had at least one child during the observation period (treated group) and 524 (21%) remained childless (control group).

While there is important attrition in the cohort study, we conducted robustness checks using the British Household Panel Survey (BHPS) and found very similar motherhood class penalty patterns, suggesting our main findings are not driven by selective attrition in the BCS70 (Supporting Information S1: Appendix F).

The outcome of interest is social class as measured by the National Statistics Socio‐economic Classification (NS‐SEC). We recoded this variable into seven main social class categories. The first class contains the Higher managerial occupations (NS‐SEC 1.1) and Higher professional occupations (NS‐SEC 1.2) (Class I). The second class contains the Lower professional occupations (Class II). The term 'professional occupations' is generally used as a collective term for both high professional (Class I) and low professional (Class II) occupations. Where results pertain only to one of these specific classes, we use the distinct terms 'high professional' or 'low professional' accordingly.

We then have the intermediate occupations (Class III), the self‐employed class (Class IV), the lower supervisory (Class V), the semi‐routine occupations (Class VI) and the routine occupations (Class VII). Finally, we added three additional categories to this measure of social class: Education, Family care (looking after home and maternity leave) and a broad category called ‘Not working’, capturing the unemployed, the disabled, retirees, etc.

For some of our analyses, we combined Class V, Class VI, and Class VII in a broad Working class, as is generally done (Rose et al. 2005). For the transition matrices and sequence plots shown in Figures 3 and 4, we combine the intermediate and self‐employed classes, and we group Education, Family care, and Not working into a single 'Not in the labour market' category.

We compared the distribution of social class in our analytical sample at age 42 to the class distribution of women aged 40–44 years old in the Annual Population Survey and in Understanding Society, and we found a very high level of comparability and consistency between the class distribution of women in our sample and the distribution of these two surveys (see Online Appendix, Section A). This gives us confidence about the representativeness of our analytical sample for the 1970 cohort of mothers in Britain.

Table 1 shows the distribution of social class comparing mothers and childless women in three age groups: 16–24, 25–39 and 40–42. We see that class differences between mothers and childless women are almost non‐existent before 25 years old. However, in the age Group 25–39 years old, we see that childless women are already in greater numbers in high professional positions, 12.7% compared to 8.1% for mothers. This is also true for low professional positions; the proportion is 39.7% for childless women compared to 30.1% for mothers. We also see that mothers are more likely to be found out of the labour market doing family care, 18.2% versus 1% for childless women. A very similar pattern appears for the age Group 40–42 years old.

**TABLE 1:** Social class distributions by age for mothers and childless women (percentages). All figures are expressed in percentages.

		Professional/Managerial				Working class			Out of labour market		
Age group		1. High	2. Low	3. Interm	4. Self‐Emp	5. Lower S	6. Semi routine	7. Routine	8. Edu	9. Care	10. Not working
16–24	Childless	2.6	16.7	19.6	1.4	4.5	11.5	7.9	29.1	0.3	6.4
16–24	Mothers	2.3	16.7	20.6	1.1	5.4	11.3	8	25.2	4.7	4.6
25–39	Childless	12.7	39.7	17.9	4.4	4.9	8.4	3.8	2	1	5.3
25–39	Mothers	8.1	30.1	16.1	4.7	4.3	10.7	3.9	1.4	18.2	2.6
40–42	Childless	14.6	39.7	14.8	6.6	4.2	8.5	3.2	0.5	1.4	6.5
40–42	Mothers	9.1	30.6	14.5	6.2	4.1	13.3	3.6	0.6	14.7	3.4

*Note:* Expressed in percentages. 1. High Professional, 2. Low Professional, 3. Intermediate, 4. Self‐Employed, 5. Lower Supervisory, 6. Semi‐Routine, 7. Routine, 8. Education, 9. Family Care, 10. Not working/Out of the Labour Market.

The two most prevalent occupational classes of women in Britain are low professional and intermediate classes. Professional occupations generally require an advanced degree and demand specialised skills. There is often a high degree of social closure and credentialism in professional occupations (Grusky and Sørensen 1998). High professional occupations have the highest level of responsibility and authority (Wright 2005). Looking at the occupations subsumed in the high professional class, we find, among other occupations, solicitors (lawyers), medical practitioners, engineers, government officials, and professors. In low‐professional occupations, we find nurses, schoolteachers, journalists, and various types of low‐level management occupations. Intermediate occupations are mainly clerical jobs (office work), white‐collar occupations with a low degree of supervision and authority and without advanced degrees required.

Lower supervisory, semi‐routine and routine social classes generally constitute what is understood to be the working class. Routine occupations are the occupations at the extreme pole of the labour contract described above, made up of cleaners, domestics, packers, warehouse workers, and waitresses. Semi‐routine occupations are made up of assistants, such as educational or care assistants, and various receptionists. The lower supervisory constitutes much of the same working‐class occupations but with a degree of supervision.

It is worth saying something about the concept of penalty in the context of social class. In the motherhood wage or earning penalty studies, the idea of penalty is straightforward because wage is a simple unidimensional scalar, low versus high penalty. However, social class is a categorical variable. The main issue is that the space becomes relative to each category (methodology detailed below). In that sense, there is no single number that could sum up the average or overall class penalty. Therefore, the penalty will be relative to each class of reference. As detailed below in the 'Regression Estimates' section, this is implemented empirically by estimating a model for the probability of being in each occupational class.

### 3.2 Analytical Strategy

To analyse the complexity of the effects of motherhood on women's social class mobility, we turn to Sequence Analysis. Sequence Analysis has been growing in popularity in sociology and has been applied to a variety of topics involving trajectories such as employment, fertility, and time use patterns (Bukodi et al. 2016; Lesnard 2008; Vagni and Cornwell 2018; Van Winkle 2018; Widmer and Ritschard 2009). It is a method well‐suited to the study of categorical longitudinal data (Billari 2001; Billari and Piccarreta 2005). Sequence Analysis has traditionally been a descriptive methodology based on a ‘holistic’ understanding of the life course (Billari 2001). In this paper, we adopt a different framework, merging sequence analysis and causal inference techniques to produce counterfactual sequences. This framework builds on earlier work about causal sequences and individual causal effects (Barban et al. 2017; Vagni and Breen 2021).

The method we propose and apply here, Individual Synthetic Sequence Matching (ISSM), builds on this tradition by integrating sequence analysis directly into a causal framework. Its name and conceptual foundation are drawn from the Synthetic Control Method developed by Abadie and Gardeazabal (2003), to estimate the aggregate counterfactual average effect where one case is treated (generally a country or region). The 'Individual' component follows the adaptation of this logic to estimate person‐specific causal effects in large surveys with many treated cases, as proposed by Vagni and Breen (2021).

Building on these foundations, ISSM offers several key advantages for life course researchers. It captures temporal specificity by accounting for the complex timing and ordering of career patterns before treatment, which in turn helps control for unobserved characteristics. Furthermore, it offers methodological flexibility, allowing researchers to leverage the vast toolkit of sequence analysis, such as dissimilarity matrices, entropy, and transitions, and combine them with traditional covariate matching for robustness. The ISSM procedure in this study is detailed below.

#### 3.2.1 Target Trial Emulation

We follow the Target Trial Emulation (TTE) framework of Hernán and Robins (2016) to enhance the transparency and rigour of our causal inference strategy. The motivation behind the TTE approach is to improve causal inference from observational data by explicitly specifying design elements commonly found in randomised controlled trials (Fu 2023).

This approach is particularly valuable when randomised controlled trials are impractical or unethical, allowing researchers to emulate the design of such trials using observational data. While TTE does not eliminate confounding bias, it addresses design‐related biases—often termed ‘self‐inflicted biases’—that can arise from ambiguous study protocols, unspecified design, or misaligned temporal frameworks (Fu 2023). The framework has demonstrated success in fields like epidemiology and is increasingly being applied in sociological research (Dickerman et al. 2019; Vagni and Breen 2021).

Traditional studies on the motherhood penalty often aggregate all childbirth events and use fixed‐effects models to estimate their impact. These studies typically analyse data spanning 1–10 years before and after childbirth, encompassing a broad age range of mothers from 16 to 50 years old.

In contrast, our study follows the TTE protocol by explicitly defining the following components: Eligibility Criteria, Outcome, Treatment Strategies/Time Zero, and Causal Contrast and Analysis. By structuring our study within the TTE framework, we aim to produce credible quasi‐experimental causal estimates regarding the impact of motherhood on women's occupational trajectories.

##### 3.2.1.1 Eligibility Criteria

Our study uses data from a British cohort study that selected all children born in a specific week across Britain (see the BCS documentation for more information). Non‐participation was addressed through appropriate weighting. All women within this cohort are included in our analysis.

##### 3.2.1.2 Outcome

The primary outcome is occupational social class, measured using the National Statistics Socio‐economic Classification (NS‐SEC).

##### 3.2.1.3 Treatment Strategies and Time Zero

The 'treatment' is defined as the occurrence of a first birth. Subsequent births are not modelled separately, as the core interest lies in the initial transition to motherhood. Consequently, any effects from higher‐order births are considered part of the total causal effect stemming from this first event. This approach frames the first birth as the start of a new causal pathway that shapes a woman's subsequent career, a pathway which includes an altered propensity to have more children.

Women who have at least one child constitute the treated group, while those who remain childless by age 46 serve as controls. The treatment period starts in the year of the first birth, with women remaining in the treated group thereafter. For each mother, we identify a control woman with matching pre‐treatment characteristics and occupational trajectories using the statistical methods detailed below. This control serves as the counterfactual trajectory, representing what would have occurred had the mother not had a first child. The treated and control units are aligned by age (by design), and the control unit is assigned a pseudo‐treatment year corresponding to the treated unit's treatment year. We explain this procedure in detail in Section C of the Online Appendix.

#### 3.2.2 Potential Outcomes Framework and Estimand

We aim to estimate the Average Treatment effect on the Treated (ATT) within the Neyman‐Rubin framework (Imbens and Rubin 2015), defined as follows:

$$E\left[\delta_{i}|D_{i}=1\right]=E\left[Y_{i}^{1}|D_{i}=1\right]-E\left[Y_{i}^{0}|D_{i}=1\right]$$

Here, $D_{i}$ represents the treatment condition (childless vs. having a child) and $Y_{i}$ is the outcome (i.e., the career or class trajectory). The individual causal effect is denoted by $\delta_{i}$ and the parameter of interest is its average among the treated. The fundamental identification issue is that $E\left[Y_{i}^{0}|D_{i}=1\right]$ is counterfactual and must be estimated. Conceptually, this term reflects the career trajectory that treated mothers would have experienced had they remained childless.

If treated women were identical to childless women in all relevant respects, we would have:

$$E\left[Y_{i}^{0}|D_{i}=1\right]=E\left[Y_{i}^{0}|D_{i}=0\right]$$

allowing us to directly compare the observed trajectories. However, because mothers and childless women may differ in ways that affect career trajectories (i.e., $E\left[Y_{i}^{0}|D_{i}=1\right]\neq E\left[Y_{i}^{0}|D_{i}=0\right]$), the raw outcomes of the childless group might not serve as a valid counterfactual. We, therefore, apply matching methods to select from the $D_{i}=0$ pool a subset of ‘matched’ non‐mothers for whom

$$E\left[Y_{i}^{0}|D_{i}=1\right]\approx E\left[Y_{i}^{0}|D_{i}=0,matched\right]$$

We then use the observed trajectories of this matched control group to estimate the counterfactual term $E\left[Y_{i}^{0}|D_{i}=1\right]$.

Because our outcome is categorical (occupational class) we operationalise the estimand as the difference in the probabilities of being in a given occupational class c at time t, conditional on treatment status. Under identification assumption, we have

$${\text{ATT}}_{c,t}=\mathrm{Pr}\left(Y_{i,t}=c|D_{i}=1\right)-\mathrm{Pr}\left(Y_{i,t}=c|D_{i}=0\right)$$

where $Y_{i,t}$ denotes the occupational class of individual i at time t and $D_{i}$ is defined such that $D_{i}=1$ if individual i had a first child and $D_{i}=0$ otherwise. ${\text{ATT}}_{c,t}$ thus captures the average difference in the proportion of women in class c at time t between mothers and their counterfactual selves had they remained childless.

##### 3.2.2.1 Empirical Estimation With Sequence Analysis

Sequence analysis is fundamentally a matching procedure that quantifies dissimilarity between sequences through operations such as insertions, deletions, and substitutions. For example, transforming the sequence ‘AAA’ into ‘AAB’ can be done by either substituting the third character or by a combination of deletion and insertion. Each operation is assigned a cost informed by social theory (Lesnard 2010). Algorithms like Optimal Matching then seek to minimise the total cost required to transform one sequence into another, thereby generating a distance measure between pairs of sequences (Studer and Ritschard 2016).

Once we compute the sequence distance between cases, we combine this measure with other fixed covariates (e.g., social origin) in a Mahalanobis matching estimation to recover valid counterfactuals (Barban et al. 2017; Ho et al. 2011).

#### 3.2.3 Two‐Stage Matching

##### 3.2.3.1 Stage One: Sequence Matching

We first use the Optimal Matching algorithm to compute a sequence distance score between each treated unit (mothers) and each control unit (childless women). Let's denote this OM distance score $S_{i}$. We also calculate two key characteristics for each individual sequence (i): the within‐sequence entropy ($R_{i}$) and the number of sequence transitions ($E_{i}$). The within‐sequence entropy quantifies the diversity or unpredictability of the different states observed within that single sequence over its duration. The number of sequence transitions provides a measure of volatility by simply counting the total number of times the state changes from one‐time point to the immediate next within that same sequence. All sequence analyses were computed using TraMineR (Gabadinho et al. 2011). These three measures quantify the similarity in life course trajectories between individuals.

##### 3.2.3.2 Stage Two: Covariate Matching

These sequence‐derived measures are then combined with a set of covariates (denoted Χ) considered as potential confounders. The identified covariates include:

- A dummy variable for social origin (indicating if the respondent grew up in a professional‐class household, i.e., at least one parent in a professional occupation),
- A dummy variable indicating whether the respondent's mother was working during the respondent's childhood,
- A categorical variable for the region of birth.

We also include work and family preferences at age 16 as a sensitivity analysis. Summary statistics for these variables can be found in the Online Appendix, Section A. Matching is performed using 1:1 Mahalanobis distance nearest neighbour matching, using the MatchIt package (Ho et al. 2011). The Mahalanobis distance between treated and control units was calculated directly using the vector of variables containing the sequence metrics ($S_{i}$; $R_{i}$; $E_{i}$) and the other covariates (Χ) for each individual.

A detailed account of the matching procedure is provided in Online Appendix, Section D. In the Online Appendix, we compare several matching estimations, and we show that the model including the sequence Optimal Matching distance, the within‐sequence entropy, the number of transitions, and the covariates described above (mother at work during childhood, social origin and region) performed best. However, we should note that we found little evidence that mothers and childless women differ in major ways regarding potential confounders. We conclude that selection into motherhood is not a major source of bias.

Table 2 displays the balance—raw average and standardised differences—of the confounders and sequence indicators. Both the sequence matching and the Mahalanobis matching procedures effectively rebalance the dataset, and no treated units were discarded, thus allowing for a valid ATT estimation.

**TABLE 2:** Covariate balance between treated (mothers) and control groups (childless women): Raw and standardised differences post‐matching.

	Treated	Controls	Avr. Diff	Std Avr. Diff
Origin professional	0.308	0.297	0.011	0.023
Mother working (age 16)	0.458	0.475	−0.017	−0.034
North	0.166	0.164	0.001	0.004
South East^a^	0.303	0.319	−0.017	−0.036
Optimal matching (normalised over 2)	0	0.412	−0.412	
Transitions	1.894	1.904	−0.01	−0.007
Within entropy	0.285	0.298	−0.013	−0.083

^a^ Other regions omitted (see Supporting Information S1: Appendix A).

#### 3.2.4 Regression Estimates

After matching, each treated unit (mother) is paired with a matched control (childless woman), and we estimate linear probability models (LPMs) on the matched dataset. These models predict the probability that an individual belongs to occupational class c at time t. Time is explicitly incorporated into the model both as a main effect and in interaction with the treatment indicator, allowing us to examine whether the effect of motherhood on occupational outcomes varies over time.

We specify the following regression model:

$$\mathrm{Pr}\left(Y_{i,t}=c\right)=\alpha +{\delta D}_{i}+\tau t+\gamma \left(D_{i}\times t\right)\;{+\varepsilon }_{it}$$

$Y_{i,t}$ is a binary indicator equal to one if individual i belongs to occupational class c at time t, and 0 otherwise, ${\delta D}_{i}$ is the treatment indicator (1 = mother, 0 = childless), τt is a set of time fixed effects, $D_{i}\times t$ is an interaction term to test whether the effect of motherhood varies over time, $\varepsilon_{it}$ is the error term. Standard errors are clustered at both the matched pair level and the individual level (see Online Appendix, Section C). This model is run separately for each occupational class c, with a dummy variable for each class, with the interaction term (γ) testing whether the motherhood penalty changes over time post‐treatment. The predicted probabilities from these dynamic models are what are visualised in Figure 2. This model allows us to estimate both the overall effect of motherhood on class trajectories and the dynamics of this effect across different time periods.

## 4 Results

**FIGURE 1:**
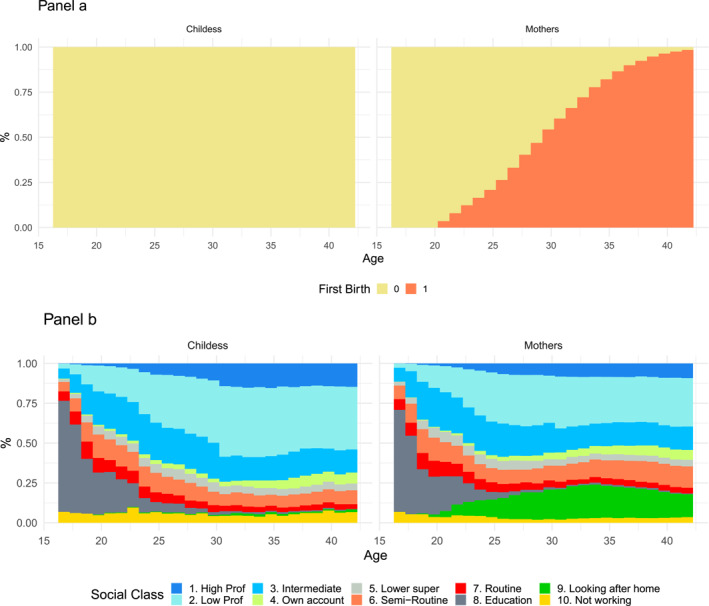
First birth timing and social class distribution by age (16–42). Panel (a) displays the timing and proportion of first births for both childless women and mothers, with age on the *x*‐axis and the proportion of first births on the *y*‐axis. Panel (b) illustrates the distribution of social classes by age, where the *x*‐axis represents age (from 16 to 42) and the *y*‐axis indicates the proportion (or frequency) of individuals in each social class.

Figure 1 provides a descriptive overview of the data. Panel A illustrates the timing of first births, showing a steady increase for mothers from their late teens to age 40, while childless women remain at zero. Panel B displays the aggregate distribution of social classes over the life course for both groups, hinting at the diverging trajectories that emerge in young adulthood, which our causal analysis aims to estimate.

**FIGURE 2:**
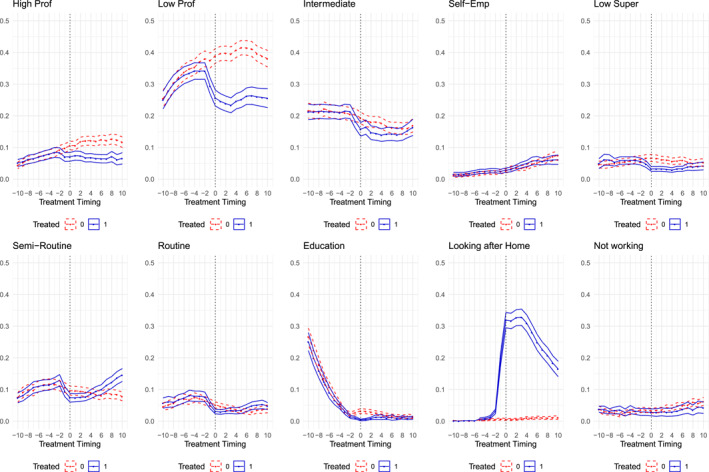
Predicted probabilities from linear models for social class trajectories. The figure displays predicted probabilities over time, ranging from 10 years before to 10 years after the first birth, based on linear probability models with clustered standard errors. The *x*‐axis shows the timing relative to the first birth, while the *y*‐axis presents the probabilities of being in each social class. Blue lines correspond to the observed trajectories of treated individuals (Treated 1), and red lines represent their estimated counterfactual trajectories (Treated 0).

Figure 2 presents the predicted probabilities from our linear probability models, following the impact of motherhood on occupational class trajectories from 10 years before to 10 years after birth. The *x*‐axis indicates time relative to birth, while the *y*‐axis displays the proportion of individuals in each social class—blue lines represent observed probabilities for mothers, and red lines depict the estimated counterfactuals. The figure highlights that the most pronounced penalties occur in both the high professional and low professional categories.

Table 3 provides the average estimates of the post‐birth penalties for each social class. These average effects are derived from a series of linear probability models estimated on the matched dataset. For each of the occupational and non‐work categories, we estimate a separate model where the dependent variable is a binary indicator for being in that class (e.g., High professional = 1), and the key independent variable is the treatment status (treated = 1 for a mother). The coefficient on the treated variable in each model thus represents the Average Treatment effect on the Treated (ATT), or the MCP, for that specific class.

**TABLE 3:** Average post‐treatment effects on occupational class trajectories. Estimates averaged from linear probability models with clustered standard errors across post‐treatment periods.

Social class	Estimate/Average (%)	Std error	*p* value
High professional	−0.048	0.016	0.0026
Low professional	−0.158	0.026	0.0000
Intermediate	−0.027	0.017	0.1191
Own account	0.007	0.01	0.4724
Lower supervisory	−0.02	0.016	0.1948
Semi‐routine	0.015	0.015	0.3097
Routine	0.001	0.008	0.8847
Education	−0.012	0.004	0.0039
Looking after home	0.261	0.008	0.0000
Not working	−0.02	0.009	0.0201

*Note:* Clustered Standard Errors (see Supporting Information S1: Appendix C).

Confirming the visual pattern, the largest MCP is found for the low professional group. Motherhood is linked to a substantial reduction, around 15 percentage points, in the likelihood that women remain in low professional occupations after birth (*p* < 0.001). This figure represents the net effect of motherhood on outcomes related to this class. It means that, overall, 15 percentage points fewer mothers are found in low professional roles compared to where they likely would have been without having children. This net difference arises from a combination of underlying shifts in mobility, such as a decrease in upward mobility into this class from lower origins, an increase in downward mobility out of this class (often into non‐employment or lower‐status work), and a reduction in the likelihood of staying within this class for those who started there.

A significant, though smaller, penalty is also observed for the high professional class, with motherhood reducing the probability of being in this top class by around 5 percentage points (*p* < 0.05). Combined, women are roughly 20 percentage points less likely to be in any professional role after becoming mothers compared to their counterfactual trajectories. Conversely, the most significant increase is seen for non‐market activity, with mothers being 26 percentage points more likely to be primarily engaged in ‘looking after home’ (*p* < 0.001) than their counterfactual selves. Other occupational classes show less pronounced average effects.

### 4.1 Transition Probabilities

**FIGURE 3:**
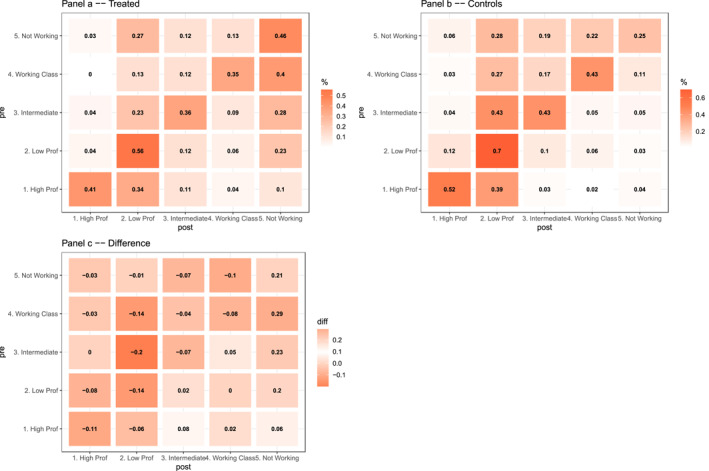
Counterfactual transition probabilities around first birth. Panel (a) displays the transition probabilities for the treated group (mothers), estimated 3 years before and 5 years after the first birth. Panel (b) shows the corresponding transition probabilities for the control group (childless women). Panel (c) presents the difference between the treated and control probabilities. In each heatmap, a darker colour indicates larger probabilities or greater differences, showing the magnitude of causal effects in transition probabilities. The probabilities are generated using the probability models as specified.

To understand how these net penalties, particularly the substantial 15 percentage points reduction for the low professional class, accumulate, we now turn to a more detailed analysis of mobility patterns. Figure 3 presents transition matrices that break down the movement between specific social class categories, comparing women's status 3 years before their first birth (Pre) to their status 5 years after (Post). This allows us to directly observe how motherhood alters the probabilities of upward mobility, downward mobility, and occupational stability for women starting from different class positions. For interpretability, we adopt a reduced class schema, grouping occupations into five broad categories: high professional, low professional, intermediate, working class, and not working, as defined in Table 1. In particular, the more granular categories, such as lower supervisory, semi‐routine, and routine occupations, are grouped under working class, following standard conventions in the reduced NS‐SEC schema.

Panel A shows the observed transitions of mothers (treated), Panel B shows the transitions for their counterfactuals (what would have happened without treatment), and Panel C highlights the difference between the two.

First, we note the high probability of the diagonal, which indicates that once a person is engaged in a class trajectory, they have a high likelihood of staying in this trajectory. This is particularly true for women engaged in professional occupations. However, comparing panel A and panel B, we see that the strength of this association is even stronger in mothers' counterfactual status.

The counterfactual immobility, staying in the same class (Panel B diagonal), shows that 70% of women starting in low professional roles would have remained there, compared to only 56% who actually did (Panel A). The difference (Panel C) is −14 percentage points, indicating reduced stability. Similarly, stability in high professional roles is reduced by 11 percentage points. This reduced stability within professional classes, especially the 14 percentage points decrease for low professionals, is a key component contributing to the overall penalties identified earlier.

Downward transitions, particularly exits from the labour market ('Not Working' ‐ Column 5), are substantially increased by motherhood. Mothers starting in working class (+29pp), intermediate (+23pp), and low professional (+20pp) occupations were significantly more likely to be 'Not Working' five years post‐birth compared to their counterfactuals. These increased exits directly correspond to the reduced probabilities of remaining in or moving up into higher occupational classes. Conversely, counterfactually, about 20 percentage points more women who were 'Not Working' pre‐birth would likely have entered the labour market (indicated by negative values summing across working categories in Row 5, Panel C).

Panel C shows that motherhood acts as a significant barrier to upward social mobility. Compared to the counterfactual, motherhood reduced the probability of women in intermediate occupations reaching low professional status by 20 percentage points (Panel C: −0.20), women in working‐class roles reaching low professional status by 14 percentage points (Panel C: −0.14), and women in low professional roles reaching high professional status by 8 percentage points (Panel C: −0.08).

### 4.2 Individual Variation by Social Class

**FIGURE 4:**
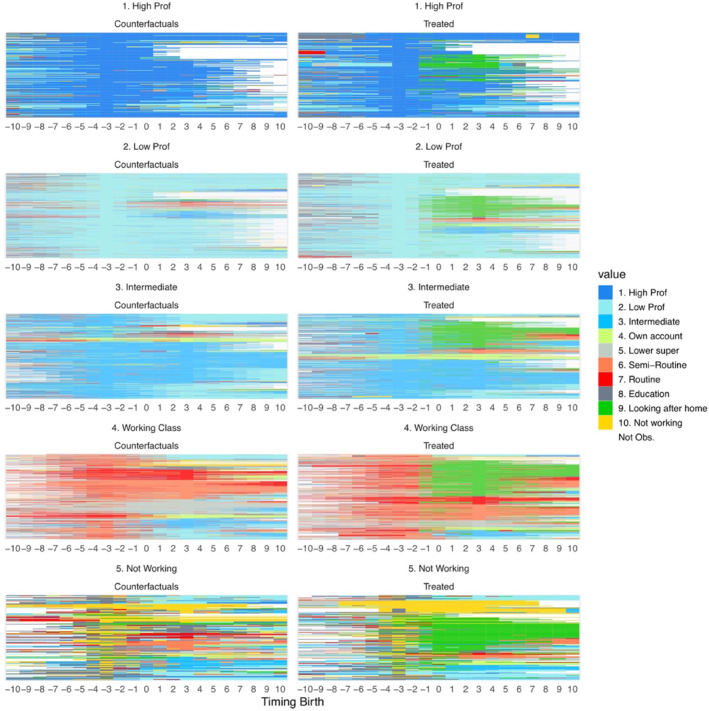
ISSM sequence index plots of individual social class trajectories for treated mothers and their counterfactuals. The figure shows the heterogeneity of women's occupational pathways beyond average effects. This plot displays individual social class sequences for treated women and their counterfactual matches, from 10 years before to 10 years after the first birth. Each horizontal line represents an individual's social class trajectory over time, showing the comparison between the observed paths of mothers and the estimated counterfactual paths of what their trajectories would have been had they remained childless. To facilitate a clear comparison, both treated mothers and their matched controls are classified according to their occupational status 3 years before the actual or assigned year of childbirth.

Sequence index plots (Gabadinho et al. 2011) reveal the heterogeneity of women's occupational pathways that would otherwise be obscured by only looking at average effects. Figure 4 displays the individual‐level class trajectories for mothers, stratified by pre‐birth social class (again using the reduced schema). Each row (horizontal line) represents one woman's social class sequence from 10 years before to 10 years after birth, showing both the observed trajectory and her counterfactual. In other words, each horizontal line represents one mother's observed trajectory (right panel) shown alongside the trajectory of her unique counterfactual match (left panel), allowing for a direct, line‐by‐line comparison between an individual and her estimated counterfactual.

Among women who began in high professional positions, motherhood creates a visible divergence from the counterfactual trajectory. Following childbirth, we observe a shift in the treated group, with fewer women maintaining their high professional status compared to their counterfactual counterparts. Some new mothers transition to lower professional roles or intermediate positions, represented by the lighter blue. Some exit the workforce entirely, indicated by the green areas absent in the counterfactual scenario.

Women in low professional positions demonstrate similar but somewhat more pronounced post‐birth transitions. There's a more substantial shift towards intermediate positions and non‐working statuses compared to high professionals. The sequence plot suggests that this group faces greater challenges in maintaining their pre‐birth occupational status, with more pronounced transitions to non‐working classifications after becoming mothers.

The intermediate group shows important contrasts between treated and counterfactual scenarios. Post‐birth, the treated panel shows a substantial increase in green areas (representing ‘Looking after home’ status), indicating that many women in intermediate occupations exit the workforce after childbirth. The counterfactual panel suggests that without motherhood, these women would have maintained more stable employment trajectories, potentially even transitioning to higher‐status positions. This group appears to experience significant occupational disruption following childbirth, with fewer returning to their previous occupational status compared to women in professional categories.

Among working‐class women, the MCP appears particularly pronounced. The post‐birth period in the treated panel shows a dramatic increase in red and green areas (representing routine work and looking after home), suggesting substantial workforce exits or downward occupational mobility. The counterfactual scenario indicates that without motherhood, these women would have maintained more consistent employment patterns. For women who were not working before childbirth, motherhood appears to extend their period of non‐employment. While their counterfactual matches show gradual transitions into employment over time (represented by shifts from yellow/green to blues and reds), the treated group demonstrates more persistent non‐working status after childbirth.

The figure highlights class‐based disparities in post‐birth labour market attachment. Across all groups, time spent in the ‘looking after home/family’ state declines over time, but high‐professional women exit this state more quickly and often avoid it altogether. By contrast, women in low‐professional and intermediate occupations frequently experience downward mobility or prolonged exits from the labour market. A particularly large share of working‐class women become, and remain, out of the labour market after childbirth.

### 4.3 Explaining Class‐Based Heterogeneity

To better understand these trajectories, Table 4 compares the background characteristics of mothers by their pre‐birth occupational class. Women in high‐professional occupations have an average age at first birth of 33 years, compared to 26 among working‐class women. We also observe strong social origin effects: about 50% of women in the high professional group had a parent in a professional occupation, compared to 20% in the working class. They were also more likely to have grown up with a working mother.

**TABLE 4:** Characteristics of mothers (treated) by social class status.

	Class pre‐treatment (3 years before birth)	Age at first birth (avr)	Social origin professional (%)	Mother working (%)	South East (%)	FT at 42 years (%)	PT at 42 years (%)
1	High professional	33	0.489	0.516	0.388	0.991	0.009
2	Low professional	31	0.376	0.495	0.309	0.884	0.116
3	Intermediate	29	0.277	0.496	0.318	0.795	0.205
4	Working class	26	0.194	0.377	0.253	0.677	0.323
5	Not working	26	0.281	0.377	0.299	0.689	0.311

*Note:* Full‐time and Part‐time are calculated for employed workers.

Regionally, high professionals are disproportionately from the South East of England, a more affluent area. Looking at long‐term labour force attachment, the last two columns of Table 4 show stark differences at age 42: high‐professional mothers are far more likely to be working full‐time, while working‐class mothers are much more likely to be in part‐time employment (32%) or out of the labour market entirely. Virtually no mothers in the high‐professional group are employed part‐time.

### 4.4 Sensitivity Analysis

We performed several sensitivity and robustness checks. All are presented in the Online Appendix F. The first major check was to reproduce our analysis on another dataset. We reproduced our analyses on the British Household Panel and found a very high degree of consistency with the results we have presented here (see Section F of the Supporting Information S1: Appendix). We find remarkably similar penalties in the two datasets. In the BHPS, we estimate the penalty for High Professional to be 3 percentage points, while in the BCS, we estimate it at 5 percentage points. For Low Professional, we estimate the penalty to be 14 percentage points with the BHPS and 16 percentage points for the BCS.

We conducted further checks using another theoretically important set of potential confounders: work preferences at age 16 and family preferences at age 16. The main reason this factor is not included in our main analysis is that it was only collected for a sub‐sample of the cohort, thus dramatically reducing the sample size (see Section D, Online Appendix). We replicated our analyses on this sub‐sample using work and family preferences indicators (Hakim 2006). While we found that preferences had explanatory power for predicting social class at age 42 years old, they were not confounders (i.e., omitting them did not create bias) but rather independent predictors. Therefore, the results including preferences in the matching do not change the results presented here (see Section D of the Online Appendix).

## 5 Discussion

Our findings reveal a substantial and enduring motherhood penalty in occupational trajectories in Britain. We estimate that motherhood lowers women's chances of reaching professional‐class occupations by approximately 20 percentage points, meaning that without this penalty, one in five more mothers would attain professional‐class status. This reflects a considerable loss of human capital for society as a whole. Motherhood reduces women's chances of advancing up the occupational hierarchy and limits their ability to return to their pre‐birth occupational class status.

These findings generally offer support to Hypothesis 1, as across all social classes, mothers are less likely to remain in the same occupational class, less likely to experience upward mobility, and more likely to experience downward mobility or exit the labour market altogether. However, our findings show that these patterns are not uniformly distributed across social classes. While professional‐class women are more likely to remain employed and retain class stability after birth, mothers in working‐class positions face precarious trajectories in terms of labour market exit and greater occupational instability, particularly in the years immediately following childbirth.

What do our findings suggest about Hypothesis 2 (professional‐class mothers face greater long‐term penalties in forgone upward mobility) and Hypothesis 3 (working‐class mothers face greater short‐term instability)? Regarding Hypothesis 2, it is mothers in low professional occupations who appear to face the most significant long‐term penalties. These women incurred large long‐term losses in upward mobility, which we might think of as foregone upward mobility. While high professional mothers also experience penalties (an average of 5 percentage points), these are smaller in magnitude and often mitigated by stronger labour market attachment and faster return to work. Moreover, low professional mothers also exhibit lower class stability, suggesting they are less likely to return to their pre‐birth occupational status compared to mothers in other classes. Turning to Hypothesis 3, working‐class mothers do show pronounced short‐term exits from employment around the time of childbirth, with marked transitions into ‘not working’ and ‘looking after home’ categories. However, this instability is often not only confined to the short term but has long‐lasting effects for these mothers. In contrast, high professional mothers show much greater short‐term stability and a quicker occupational recovery. It is also important to note that a substantial share of working‐class women would have moved into low professional occupations had they not become mothers. This further highlights the long‐term opportunity costs of motherhood for women across the occupational spectrum, particularly in terms of forgone upward mobility.

In conclusion, we can say that Hypothesis 2 is partially supported when focusing specifically on low professional women, who demonstrate significant long‐term penalties through substantial foregone upward mobility compared to their counterfactual trajectories. Hypothesis 3 is supported as working‐class women experience greater short‐term occupational instability following childbirth, with pronounced transitions to non‐working statuses and limited returns to previous employment levels.

We have shown that these class‐based differences reflect deeper structural inequalities in mothers' life courses. Professional‐class mothers tend to be older at the time of first birth, better educated, and more embedded in favourable regional labour markets (such as the South East). They are also more likely to benefit from stronger intergenerational support, evidenced by higher social class of origin and higher rates of maternal employment in their family backgrounds. These cumulative advantages appear to buffer them against some of the harsher long‐term occupational penalties associated with motherhood. By contrast, working‐class mothers are more likely to have children at younger ages, to experience discontinuous employment histories, and to return to work in part‐time jobs after birth. They are also more likely to come from disadvantaged social origins, further exacerbating the challenges they face in maintaining or advancing their occupational status post‐childbirth. Overall, our findings support and extend earlier work (e.g., Dex et al. 2008) by showing that motherhood plays an important role in shaping women's long‐term occupational mobility, limiting the opportunities of those already in disadvantaged class positions prior to childbirth, and thus reinforcing social inequalities.

Several limitations of this study should be acknowledged. While we have employed the standard UK social class classification, its broad level of aggregation may obscure important within‐class differences and other relevant dimensions of stratification. Future research could explore alternative class schemas that capture variations in authority, work schedules, or job prestige. As with all longitudinal analyses, sample‐related issues such as attrition and censoring may introduce bias or limit the representativeness of the results. Another key limitation is the absence of partner trajectories. Decisions around employment and caregiving are typically made within households, and future work should incorporate partner careers to better understand how couples navigate occupational trade‐offs after childbirth. Moreover, while this paper provides robust causal estimates of the motherhood class penalty, it does not unpack the mechanisms through which these penalties emerge. Future research should investigate how individual preferences, occupational structures, and institutional constraints interact to shape mothers' career paths.

Another limitation is that our analysis, using the standard NS‐SEC classification, does not differentiate between full‐time and part‐time employment. Methodologically, one cannot simply control for post‐birth employment status, as a switch to part‐time work is often a direct consequence of motherhood and thus a central mechanism through which the class penalty operates. Future work could address this by developing a form of causal mediation analysis, which would enable the decomposition of the total Motherhood Class Penalty and the quantification of the extent to which it is mediated by a transition to part‐time work versus other factors.

Finally, our findings are based on a single historical context, the 1970 British birth cohort. Future studies should examine whether the magnitude and shape of the motherhood class penalty differ across cohorts and countries. Patterns of class mobility may look markedly different under varying welfare regimes and labour market structures, such as those found in Scandinavian countries, Germany, France, or the United States.

In this paper, using a novel method we call Individual Synthetic Sequence Matching (ISSM), we introduced and documented what we call the Motherhood Class Penalty (MCP), a persistent and substantial occupational disadvantage experienced by women following childbirth. Our findings show that the transition to motherhood imposes a significant cost on women's long‐term class mobility, primarily by restricting upward progression and increasing the likelihood of downward movement or labour market exit. This penalty particularly hinders upward mobility for working‐class women and ultimately reinforces intra‐generational inequality.

## Conflicts of Interest

The author declares no conflicts of interest.

## Supporting information


Supporting Information S1

## Data Availability

The data that support the findings of this study are openly available in UK Data Service at DOI: http://doi.org/10.5255/UKDA‐Series‐200001, reference number SN: 200001.

## References

[bjos70039-bib-0001] Abadie, A. , and J. Gardeazabal . 2003. “The Economic Costs of Conflict: A Case Study of the Basque Country.” American Economic Review 93, no. 1: 113–132. 10.1257/000282803321455188.

[bjos70039-bib-0002] Abendroth, A.‐K. , M. L. Huffman , and J. Treas . 2014. “The Parity Penalty in Life Course Perspective: Motherhood and Occupational Status in 13 European Countries.” American Sociological Review 79, no. 5: 993–1014. 10.1177/0003122414545986.

[bjos70039-bib-0003] Aisenbrey, S. , M. Evertsson , and D. Grunow . 2009. “Is There a Career Penalty for Mothers’ Time Out? A Comparison of Germany, Sweden and the United States.” Social Forces 88, no. 2: 573–605. 10.1353/sof.0.0252.

[bjos70039-bib-0004] Albrecht, J. , A. Björklund , and S. Vroman . 2003. “Is There a Glass Ceiling in Sweden?” Journal of Labor Economics 21, no. 1: 145–177. 10.1086/344126.

[bjos70039-bib-0005] Altintas, E. , and O. Sullivan . 2017. “Trends in Fathers’ Contribution to Housework and Childcare Under Different Welfare Policy Regimes.” Social Politics: International Studies in Gender, State & Society 24, no. 1: 81–108. 10.1093/sp/jxw007.

[bjos70039-bib-0006] Anderson, D. J. , M. Binder , and K. Krause . 2002. “The Motherhood Wage Penalty: Which Mothers Pay it and Why?” American Economic Review 92, no. 2: 354–358. 10.1257/000282802320191606.

[bjos70039-bib-0007] Andrew, A. , O. Bandiera , M. Costa Dias , et al. 2021. The Careers and Time Use of Mothers and Fathers, Vol. 319. IFS Briefing Note.

[bjos70039-bib-0008] Barban, N. , X. de Luna , E. Lundholm , I. Svensson , and F. C. Billari . 2017. “Causal Effects of the Timing of Life‐Course Events: Age at Retirement and Subsequent Health.” Sociological Methods & Research 49, no. 1: 216–249. 10.1177/0049124117729697.

[bjos70039-bib-0009] Billari, F. C. 2001. “The Analysis of Early Life Courses: Complex Descriptions of the Transition to Adulthood.” Journal of Population Research 18, no. 2: 119–142. 10.1007/bf03031885.

[bjos70039-bib-0010] Billari, F. C. , and R. Piccarreta . 2005. “Analyzing Demographic Life Courses Through Sequence Analysis.” Mathematical Population Studies 12, no. 2: 81–106. 10.1080/08898480590932287.

[bjos70039-bib-0011] Bryson, A. , H. Joshi , B. Wielgoszewska , and D. Wilkinson . 2020. “A Short History of the Gender Wage Gap in Britain.” Oxford Review of Economic Policy 36, no. 4: 836–854. 10.1093/oxrep/graa046.

[bjos70039-bib-0012] Budig, M. J. , and P. England . 2001. “The Wage Penalty for Motherhood.” American Sociological Review 66, no. 2: 204–225. 10.1177/000312240106600203.

[bjos70039-bib-0013] Bukodi, E. , and S. Dex . 2010. “Bad Start: Is There a Way Up? Gender Differences in the Effect of Initial Occupation on Early Career Mobility in Britain.” European Sociological Review 26, no. 4: 431–446. 10.1093/esr/jcp030.

[bjos70039-bib-0014] Bukodi, E. , and J. H. Goldthorpe . 2018. Social Mobility and Education in Britain. Cambridge University Press.

[bjos70039-bib-0015] Bukodi, E. , J. H. Goldthorpe , B. Halpin , and L. Waller . 2016. “Is Education now Class Destiny? Class Histories Across Three British Birth Cohorts.” European Sociological Review 32, no. 6: 835–849. 10.1093/esr/jcw041.

[bjos70039-bib-0016] Bukodi, E. , J. H. Goldthorpe , H. Joshi , and L. Waller . 2017. “Why Have Relative Rates of Class Mobility Become More Equal Among Women in Britain?” British Journal of Sociology 68, no. 3: 512–532. 10.1111/1468-4446.12274. 28700076

[bjos70039-bib-0017] Correll, S. J. , S. Benard , and I. Paik . 2007. “Getting a Job: Is There a Motherhood Penalty?” American Journal of Sociology 112, no. 5: 1297–1339. 10.1086/511799.

[bjos70039-bib-0018] Costa Dias, M. , R. Joyce , and F. Parodi . 2020. “The Gender Pay Gap in the UK: Children and Experience in Work.” Oxford Review of Economic Policy 36, no. 4: 855–881. 10.1093/oxrep/graa053.

[bjos70039-bib-0019] Cukrowska‐Torzewska, E. , and A. Matysiak . 2020. “The Motherhood Wage Penalty: A Meta‐Analysis.” Social Science Research 88: 102416. 10.1016/j.ssresearch.2020.102416. 32469733

[bjos70039-bib-0020] Davies, R. , and G. Pierre . 2005. “The Family Gap in Pay in Europe: A Cross‐Country Study.” Labour Economics 12, no. 4: 469–486. 10.1016/j.labeco.2005.05.003.

[bjos70039-bib-0021] de Linde Leonard, M. , and T. D. Stanley . 2020. “The Wages of Mothers’ Labor: A Meta‐Regression Analysis.” Journal of Marriage and Family 82, no. 5: 1534–1552. 10.1111/jomf.12693.

[bjos70039-bib-0022] Dex, S. 1987. Women’s Occupational Mobility: A Lifetime Perspective. Springer.

[bjos70039-bib-0023] Dex, S. , and E. Bukodi . 2012. “The Effects of Part‐Time Work on Women’s Occupational Mobility in Britain: Evidence From the 1958 Birth Cohort Study.” National Institute Economic Review 222: R20–R37. 10.1177/002795011222200103.

[bjos70039-bib-0024] Dex, S. , K. Ward , and H. Joshi . 2008. “Changes in Women’s Occupations and Occupational Mobility over 25 Years.” In Women and Employment: Changing Lives and New Challenges, edited by J. L. Scott , S. Dex , and H. Joshi , 54–80. Edward Elgar Publishing.

[bjos70039-bib-0025] Dickerman, B. A. , X. García‐Albéniz , R. W. Logan , S. Denaxas , and M. A. Hernán . 2019. “Avoidable Flaws in Observational Analyses: An Application to Statins and Cancer.” Nature medicine 25, no. 10: 1601–1606. 10.1038/s41591-019-0597-x. PMC7076561 31591592

[bjos70039-bib-0026] England, P. , J. Bearak , M. J. Budig , and M. J. Hodges . 2016. “Do Highly Paid, Highly Skilled Women Experience the Largest Motherhood Penalty?” American Sociological Review 81, no. 6: 1161–1189. 10.1177/0003122416673598.

[bjos70039-bib-0027] Erikson, R. , and J. H. Goldthorpe . 1992. The Constant Flux: A Study of Class Mobility in Industrial Societies. Clarendon Press.

[bjos70039-bib-0028] Esping‐Andersen, G. 1990. The Three Worlds of Welfare Capitalism. Polity Press.

[bjos70039-bib-0029] Francis‐Devine, B. , K. Zaidi , and A. Murray . 2025. Women and the UK Economy (No. 6838). House of Commons Library.

[bjos70039-bib-0030] Fu, E. L. 2023. “Target Trial Emulation to Improve Causal Inference From Observational Data: What, Why, and How?” Journal of the American Society of Nephrology 34, no. 8: 1305–1314. 10.1681/asn.0000000000000152. 37131279 PMC10400102

[bjos70039-bib-0031] Gabadinho, A. , G. Ritschard , N. S. Mueller , and M. Studer . 2011. “Analyzing and Visualizing State Sequences in R With TraMineR.” Journal of Statistical Software 40, no. 4: 1–37. 10.18637/jss.v040.i04.

[bjos70039-bib-0032] Gangl, M. , and A. Ziefle . 2009. “Motherhood, Labor Force Behavior, and Women’s Careers: An Empirical Assessment of the Wage Penalty for Motherhood in Britain, Germany, and the United States.” Demography 46, no. 2: 341–369. 10.1353/dem.0.0056. 21305397 PMC2831275

[bjos70039-bib-0033] Goldin, C. 2006. “The Quiet Revolution That Transformed Women’s Employment, Education, and Family.” American Economic Review 96, no. 2: 1–21. 10.1257/000282806777212350.

[bjos70039-bib-0034] Goldin, C. 2021. Career and Family: Women’s Century‐Long Journey Toward Equity. Princeton University Press.

[bjos70039-bib-0035] Gough, M. , and M. Noonan . 2013. “A Review of the Motherhood Wage Penalty in the United States.” Sociology Compass 7, no. 4: 328–342. 10.1111/soc4.12031.

[bjos70039-bib-0036] Grusky, D. B. , and J. B. Sørensen . 1998. “Can Class Analysis be Salvaged?” American Journal of Sociology 103, no. 5: 1187–1234. 10.1086/231351.

[bjos70039-bib-0037] Hakim, C. 2006. “Women, Careers, and Work‐Life Preferences.” British Journal of Guidance and Counselling 34, no. 3: 279–294. 10.1080/03069880600769118.

[bjos70039-bib-0038] Harkness, S. , M. Borkowska , and A. Pelikh . 2019. Employment Pathways and Occupational Change After Childbirth. UK Government Equalities Office.

[bjos70039-bib-0039] Harkness, S. , and J. Waldfogel . 2003. “The Family Gap in Pay: Evidence From Seven Industrialized Countries.” Research in Labor Economics 22: 369–413. 10.1016/s0147-9121(03)22012-4.

[bjos70039-bib-0040] Hernán, M. A. , and J. M. Robins . 2016. “Using Big Data to Emulate a Target Trial When a Randomized Trial Is Not Available.” American Journal of Epidemiology 183, no. 8: 758–764. 10.1093/aje/kwv254. 26994063 PMC4832051

[bjos70039-bib-0041] Ho, D. E. , G. King , and E. A. Stuart . 2011. “Matchit: Nonparametric Preprocessing for Parametric Causal Inference.” Journal of Statistical Software 42, no. 8: 1–28. 10.18637/jss.v042.i08.

[bjos70039-bib-0042] Icardi, R. , A. E. Hägglund , and M. Fernández‐Salgado . 2022. “Fatherhood and Wage Inequality in Britain, Finland, and Germany.” Journal of Marriage and Family 84, no. 1: 273–290. 10.1111/jomf.12792. 35874105 PMC9292225

[bjos70039-bib-0043] Imbens, G. W. , and D. B. Rubin . 2015. Causal Inference in Statistics, Social, and Biomedical Sciences. Cambridge University Press.

[bjos70039-bib-0044] Ishizuka, P. 2021. “The Motherhood Penalty in Context: Assessing Discrimination in a Polarized Labor Market.” Demography 58, no. 4: 1275–1300. 10.1215/00703370-9373587. 34236402

[bjos70039-bib-0045] Jacobs, S. 1999. “Trends in Women’s Career Patterns and in Gender Occupational Mobility in Britain.” Gender, Work and Organization 6, no. 1: 32–46. 10.1111/1468-0432.00067.

[bjos70039-bib-0046] Joshi, H. , and P. A. Hinde . 1993. “Employment After Childbearing in Post‐War Britain: Cohort‐Study Evidence on Contrasts Within and Across Generations.” European Sociological Review 9, no. 3: 203–227. 10.1093/oxfordjournals.esr.a036678. 12288853

[bjos70039-bib-0047] Kalabikhina, I. E. , P. O. Kuznetsova , and S. A. Zhuravleva . 2024. “Size and Factors of the Motherhood Penalty in the Labour Market: A Meta‐Analysis.” Population and Economics 8, no. 2: 178–205. 10.3897/popecon.8.e121438.

[bjos70039-bib-0048] Kleven, H. , C. Landais , and J. E. Søgaard . 2019. “Children and Gender Inequality: Evidence From Denmark.” American Economic Journal: Applied Economics 11, no. 4: 181–209. 10.1257/app.20180010.

[bjos70039-bib-0049] Leoncini, R. , M. Macaluso , and A. Polselli . 2024. “Gender Segregation: Analysis Across Sectoral Dominance in the UK Labour Market.” Empirical Economics 67, no. 5: 2289–2343. 10.1007/s00181-024-02611-1.

[bjos70039-bib-0050] Lesnard, L. 2008. “Off‐Scheduling Within Dual‐Earner Couples: An Unequal and Negative Externality for Family Time.” American Journal of Sociology 114, no. 2: 447–490. 10.1086/590648.

[bjos70039-bib-0051] Lesnard, L. 2010. “Setting Cost in Optimal Matching to Uncover Contemporaneous Socio‐Temporal Patterns.” Sociological Methods & Research 38, no. 3: 389–419. 10.1177/0049124110362526.

[bjos70039-bib-0052] Mari, G. 2019. “Is There a Fatherhood Wage Premium? A Reassessment in Societies With Strong Male‐Breadwinner Legacies.” Journal of Marriage and Family 81, no. 5: 1033–1052. 10.1111/jomf.12600.

[bjos70039-bib-0053] Musick, K. , M. D. Bea , and P. Gonalons‐Pons . 2020. “His and Her Earnings Following Parenthood in the United States, Germany, and the United Kingdom.” American Sociological Review 85, no. 4: 639–674. 10.1177/0003122420934430.

[bjos70039-bib-0054] Office for National Statistics . 2023. “NS‐SEC, Census 2021.” https://www.ons.gov.uk/datasets/TS062/editions/2021/versions/5.

[bjos70039-bib-0055] Rose, D. , D. J. Pevalin , and K. O’Reilly . 2005. The National Statistics Socio‐Economic Classification: Origins, Development and Use. Palgrave Macmillan.

[bjos70039-bib-0056] Schmidt, V. A. 2002. The Futures of European Capitalism. Oxford University Press.

[bjos70039-bib-0057] Studer, M. , and G. Ritschard . 2016. “What Matters in Differences Between Life Trajectories: A Comparative Review of Sequence Dissimilarity Measures.” Journal of the Royal Statistical Society: Series A 179, no. 2: 481–511. 10.1111/rssa.12125.

[bjos70039-bib-0058] UCL Social Research Institute . 2025. 1970 British Cohort Study (SN: 200001). UK Data Service. 10.5255/UKDA-Series-200001.

[bjos70039-bib-0059] University College London, UCL Institute of Education, Centre for Longitudinal Studies . 2023. 1970 British Cohort Study: Activity Histories, 1986‐2016. UK Data Service. SN: 8787. 10.5255/UKDA-SN-8787-1.

[bjos70039-bib-0060] Vagni, G. , and R. Breen . 2021. “Earnings and Income Penalties for Motherhood: Estimates for British Women Using the Individual Synthetic Control Method.” European Sociological Review 37, no. 5: 834–848. 10.1093/esr/jcab014.

[bjos70039-bib-0061] Vagni, G. , and B. Cornwell . 2018. “Patterns of Everyday Activities Across Social Contexts.” Proceedings of the National Academy of Sciences 115, no. 24: 6183–6188. 10.1073/pnas.1718020115. PMC6004479 29848627

[bjos70039-bib-0062] Van Winkle, Z. 2018. “Family Trajectories Across Time and Space: Increasing Complexity in Family Life Courses in Europe?” Demography 55, no. 1: 135–164. 10.1007/s13524-017-0628-5. 29255975

[bjos70039-bib-0063] Waldfogel, J. 1997. “The Effect of Children on Women’s Wages.” American Sociological Review 62, no. 2: 209–217. 10.2307/2657300.

[bjos70039-bib-0064] Weeden, K. A. , and D. B. Grusky . 2005. “The Case for a New Class Map.” American Journal of Sociology 111, no. 1: 141–212. 10.1086/428815.

[bjos70039-bib-0065] Widmer, E. D. , and G. Ritschard . 2009. “The De‐Standardization of the Life Course: Are Men and Women Equal?” Advances in Life Course Research 14, no. 1: 28–39. 10.1016/j.alcr.2009.04.001.

[bjos70039-bib-0066] Wilde, E. T. , L. Batchelder , and D. T. Ellwood . 2010. The Mommy Track Divides: The Impact of Childbearing on Wages of Women of Differing Skill Levels. National Bureau of Economic Research. Accessed, April 7, 2025. https://www.nber.org/papers/w16582.

[bjos70039-bib-0067] Wright, E. O. 2005. Approaches to Class Analysis. Cambridge University Press.

