# Supplementary material for: Life Course Social Mobility and Parenthood. Counterfactual Estimates of the Motherhood Class Penalty in Britain
Source: Br J Sociol. 2025 Oct 21;77(1):114–29. doi: 10.1111/1468-4446.70039 (PMC12793722; doi:10.1111/1468-4446.70039)
Supplement: Supplementary file 1 — Supporting Information S1 [file BJOS-77-114-s001.pdf]

Online Appendix

Life Course Social Mobility and Parenthood. Counterfactual Estimates of the Motherhood Class Penalty in Britain

Contents

Appendices **1**

A BCS Data Sample **3**
A.1 Sample . . . 3
A.1.1 Comparability/Representativity . . . 3
A.1.2 Treatment . . . 4
A.1.3 Social Class Operationalisation . . . 4
A.2 Characteristics at Age 16 years . . . 7
A.2.1 Potential Treatment . . . 9
A.2.2 Sub-Sample Preferences . . . 11

B DAG Model **13**

C Data Structure **15**
C.1 Introduction . . . 15
C.1.1 Individual Estimates . . . 16
C.2 Modeling . . . 16
C.2.1 Mahalanobis Distance Matching Specification . . . 18
C.2.2 Estimating the Treatment Effect . . . 18
C.2.3 Standard Errors . . . 19

D Matching Strategy **21**
D.1 Matching Quality . . . 21
D.1.1 Models . . . 21

E The Potential Outcomes Framework **29**
E.1 Introduction . . . 29
E.1.1 Average and Individual Effects . . . 30

F BHPS **33**
F.1 Sample Selection . . . 34
F.2 Main Results BHPS . . . 35

G Clusters **37**
G.1 SeqIplot 37
G.2 Cluster Working Class 43

List of Figures

A.1 Proportion of Birth by Age for Men and Women 5
A.2 Social Class by Treated (Mothers) and Controls (Childless) **Monthly** – 1986 to 2012 6
A.3 Proportion of Missing Cases by Treatment Timing Sequence Distribution Plot 10

B.1 Theoretical DAG 13

D.1 Covariate Balance Before and After Matching for Full Sample and Preference Sample 26
D.2 Covariate Balance Before and After Matching for Full Sample and Preference Sample. Outcomes. 27

F.1 BHPS Average Class Outcomes 36

G.1 Sequence Index Plot of Class Trajectories for Women Starting in Class 1. Each line represents an individual trajectory from 3 years before birth (3) to 10 years after birth (0 = year of birth). The vertical axis displays class membership, while the horizontal axis indicates time relative to childbirth. Trajectories are aligned at year 0 to highlight patterns before and after birth. 38
G.2 Sequence Index Plot of Class Trajectories for Women Starting in Class 2. Each line represents an individual trajectory from 3 years before birth (3) to 10 years after birth (0 = year of birth). The vertical axis displays class membership, while the horizontal axis indicates time relative to childbirth. Trajectories are aligned at year 0 to highlight patterns before and after birth. 39

G.3 Sequence Index Plot of Class Trajectories for Women Starting in Class 3. Each line represents an individual trajectory from 3 years before birth (3) to 10 years after birth (0 = year of birth). The vertical axis displays class membership, while the horizontal axis indicates time relative to childbirth. Trajectories are aligned at year 0 to highlight patterns before and after birth. 40
G.4 Sequence Index Plot of Class Trajectories for Women Starting in Class 6. Each line represents an individual trajectory from 3 years before birth (3) to 10 years after birth (0 = year of birth). The vertical axis displays class membership, while the horizontal axis indicates time relative to childbirth. Trajectories are aligned at year 0 to highlight patterns before and after birth. 41
G.5 Sequence Index Plot of Class Trajectories for Women Starting in Class 8-9-10. Each line represents an individual trajectory from 3 years before birth (3) to 10 years after birth (0 = year of birth). The vertical axis displays class membership, while the horizontal axis indicates time relative to childbirth. Trajectories are aligned at year 0 to highlight patterns before and after birth. 42
G.6 Pre-Treatment Working Class Clustering 44

List of Tables

A.1 NS-SEC Distribution for sample of BCS 70 (Women Age 42), Annual Population Survey and Understanding Society (Women Age 40-44 in 2012) . . . 4
A.2 Proportion of Treated Cases . . . 4
A.3 Distribution of Age at First Birth . . . 4
A.4 Outcome . . . 7
A.5 (Future) Mothers and Childless Women by Social Origin . . . 7
A.6 Future Mothers and Childless Women by Regions . . . 8
A.7 Proportion of Missing Cases by Treatment Timing . . . 9
A.8 Sub-Sample Preferences N for Treated and Controls . . . 11

C.1 text . . . 15
C.2 Example Data Structure - Wide Format I (pers id in column) . . . 16
C.3 Example Data Structure - Wide Format II . . . 16
C.4 Example Data Structure for Individual Estimation (Treated Pool) . 17
C.5 Data Structure for Final Matching . . . 17

D.1 Covariate Balance Before and After Matching for Full Sample . . . 24
D.2 Covariate Balance Before and After Matching for Preference Sub-Sample . . . 25
D.3 OLS Regression Predicting Social Class at Age 42 . . . 28

F.1 Treatment and Timing Selection . . . 34
F.2 Descriptive Statistics. N Treated and Controls. . . . 34
F.3 Pre-Treatment Characteristics Comparison between Treated (1) and Controls (0) . . . 35

Appendices

Appendix A

BCS Data Sample

A.1 Sample

The class sequence trajectories come from the harmonised "BCS70 Activity Histories Data File", which includes Age 42 Sweep 9 (2012). Based on this file we create a yearly sequence file of class trajectories taking social class and employment status in the month of October of each year. However, to make sure to use the full fertility trajectory, we use the fertility history at age 46 from Sweep 10 (2016). Unfortunately, we found impossible to construct a social class measure for the Sweep 10 to comparable to previous Sweep.

The sample is restricted to all individuals present at Sweep 9 and 10 and to all individuals that participated to the 1986 (16 years old) Sweep (which is 88% of respondents).

We also removed women who gave birth before their 20th birthday because we need at least 4 pre-treatment periods to match on. The analytical sample is a total 2541 women observed in all Sweeps from 1986 to 2012.

A.1.1 Comparability/Representativity

Question of representativity is always an issue with follow-up studies. To address this question, we compared the social class distribution of women in the BCS 70 at age 42 (in 2012) to the class distribution of women aged 40-44 in 2012 in the Understanding Society survey and the Annual Population Survey. The results are detailed below in Table A.1.

Table A.1: NS-SEC Distribution for sample of BCS 70 (Women Age 42), Annual Population Survey and Understanding Society (Women Age 40-44 in 2012)

	nssec	BCS70	APS	UKLS
1	1. High professional	0.13	0.13	0.11
2	2. Lower professional	0.37	0.32	0.35
3	3. Intermediate	0.18	0.22	0.19
4	4. Own account workers	0.07	0.07	0.07
5	5. Lower supervisory	0.05	0.04	0.04
6	6. Semi-Routine	0.15	0.15	0.20
7	7. Routine	0.04	0.07	0.05

A.1.2 Treatment

The treatment (i.e. having a child) is coded 1 whether a woman had a child before her 46th birthday.

Table A.2: Proportion of Treated Cases

	treated	n	p
1	0.00	524	0.20
2	1.00	2017	0.80

Table A.3: Distribution of Age at First Birth

	Age at first birth grouped	n	p
1	20-24	420	0.17
2	25-29	676	0.27
3	30-34	561	0.22
4	35-38	254	0.10
5	39-42	106	0.04

A.1.3 Social Class Operationalisation

For Sweep 1 to 9 we constructed the NS-SEC using employment status (JEMPST) and occupation (J90SOC). We added to the NSSEC schema the categories of the variables 'JACTIV'.

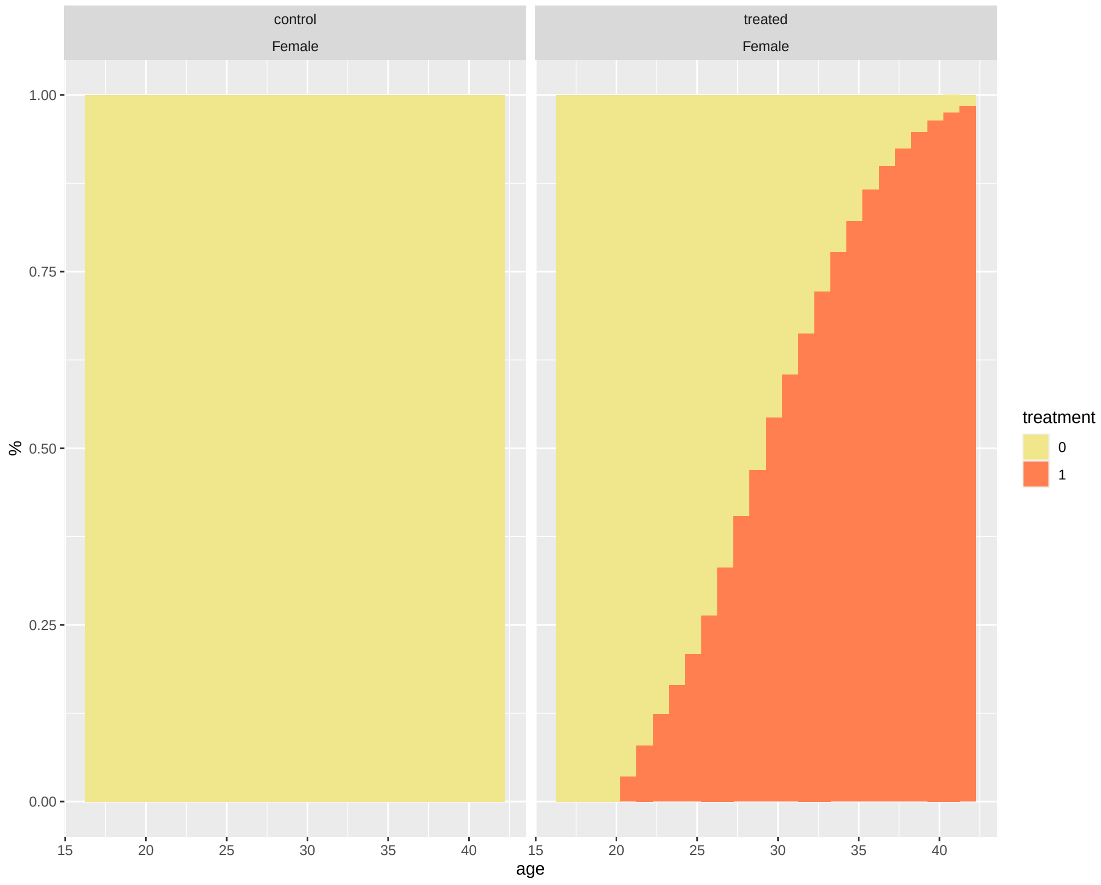

Figure A.1: Proportion of Birth by Age for Men and Women

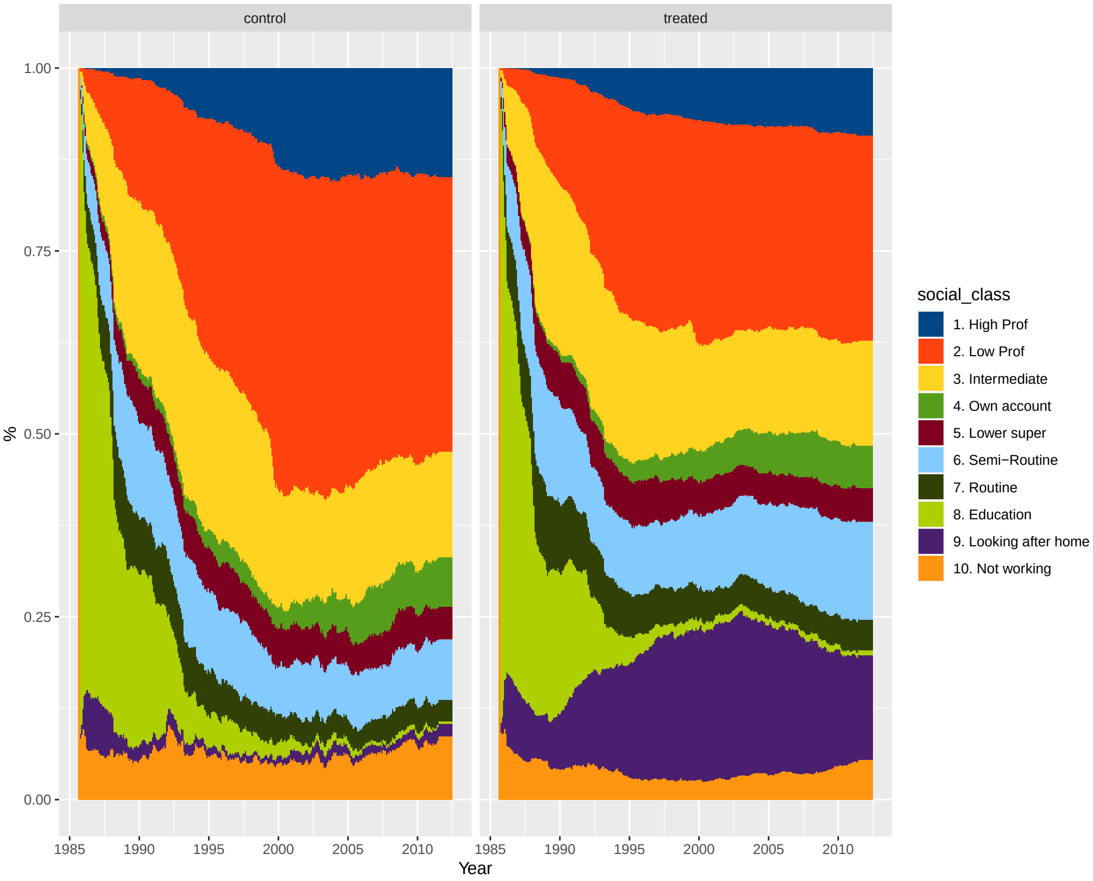

Figure A.2: Social Class by Treated (Mothers) and Controls (Childless) **Monthly** – 1986 to 2012

Table A.4: Outcome

Social Class	
1. High Professional	
2. Low Professional	
3. Intermediate	
4. Own Account	
5. Lower Super	
6. Semi-Routine	
7. Routine	
8. Education	(whether the respondent is in full-time or part-time education)
9. Looking after home	(Looking after home/family and Maternity leave)
10. Not working	(Seeking work, disabled, retired, volunteering, . . .)

A.2 Characteristics at Age 16 years

We use several variables from the 1986 Sweep when the respondent was 16, such as parental class, region of birth, work and family preferences and education indicators.

Table A.5: (Future) Mothers and Childless Women by Social Origin

Treated	Origin Professional	Mother Working
Childess	0.323	0.521
Mothers	0.318	0.472
Difference	0.005	0.049

Table A.6: Future Mothers and Childless Women by Regions

Treated	North	York	East Midlands	East Anglia	South East	South West	West Midlands	North West	Wales	Scotland	<NA>
Childess	0.063	0.101	0.078	0.034	0.277	0.101	0.101	0.107	0.036	0.090	0.011
Mothers	0.069	0.097	0.060	0.041	0.277	0.091	0.086	0.120	0.058	0.084	0.017

A.2.1 Potential Treatment

Because the timing of birth varies between mothers, the length of observation pre and post treatment will vary between mothers.

Table A.7 and Figure A.3 show the number of observed cases for different timing period going from -10 years before birth to +10 after birth.

Table A.7: Proportion of Missing Cases by Treatment Timing

	Treatment Timing	Not Observed	Cases
1	-10	0.25	0.75
2	-9	0.22	0.78
3	-8	0.20	0.80
4	-7	0.18	0.82
5	-6	0.16	0.84
6	-5	0.14	0.86
7	-4		1.00
8	-3		1.00
9	-2		1.00
10	-1		1.00
11	0		1.00
12	1	0.14	0.86
13	2	0.15	0.85
14	3	0.16	0.84
15	5	0.19	0.81
16	6	0.21	0.79
17	7	0.24	0.76
18	8	0.26	0.74
19	9	0.30	0.70
20	10	0.34	0.66

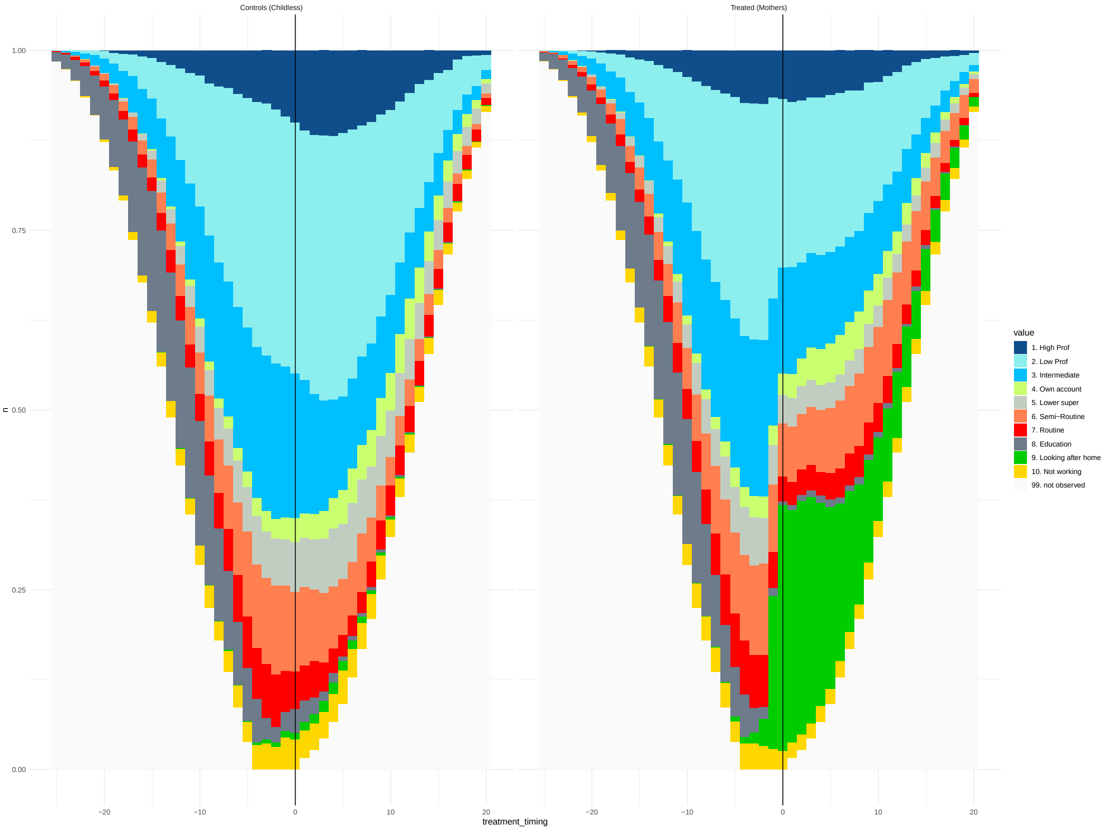

Figure A.3: Proportion of Missing Cases by Treatment Timing Sequence Distribution Plot

A.2.2 Sub-Sample Preferences

Questions related to work and family preferences were only collected for a sub-sample of the cohort at age 16 years old. As displayed in Table A.8 we can see that the sample size drops dramatically. We decided to only use this sub-sample for sensitivity analyses. We present them in Appendix Section D.

Table A.8: Sub-Sample Preferences N for Treated and Controls

	treated	n
1	0	297
2	1	951

We use two main variables as indicators of family and work preferences.

Family preferences. Question: “Interested in family life”, with (1) Very interested, (2) Quite Interested, (3) Not sure, (4) Not interested.

Work preferences. Question: “Job: To have high earnings / wages”, with (1) Matters very much, (2) Matters somewhat, (3) Doesn’t matter.

Appendix B

DAG Model

Let D be the treatment (mother vs childless), Y_1 the class trajectory after birth, and Y_0 the class trajectory before birth. Let X be the set of confounders listed below.

- Region birth
- Mother working when 16 years old
- Social Origin (parental class of origin)
- Family Preference at age 16 years old
- Work Preference at age 16 years old

Figure B.1: Theoretical DAG

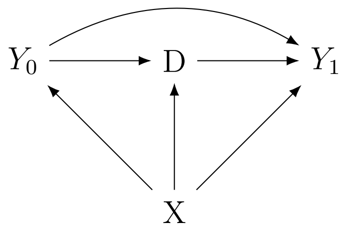

Appendix C

Data Structure

C.1 Introduction

The main issue of estimating treatment effects in longitudinal observational settings, where the treatment can occur at any time, is that there is no natural way to define when the control group should be considered "treated." Consider the following example with five units, of which two are treated.

In Table C.1 we see that unit 1 and unit 3 are the treated cases.

Table C.1: text

	id	treated_case	age treat
1	11	1	30
2	23	0	NA
3	44	1	25
4	56	0	NA
5	79	0	NA

In Table C.2, we consider the timing of the treatment and the subsequent outcome (y). The columns indicate the values for each case (e.g., treat.1 refers to case 1). We have three age periods—20, 25, and 30. Here, treated case 1 receives treatment at age 30, and treated case 2 at age 25. The outcome vector (y) indicates the social class (A, B, or C).

Because the treatment timing is unique to each treated case, estimation becomes challenging. One might attempt to estimate the effects at each age separately, as shown in Table C.3. However, this approach is cumbersome, especially when there are yearly age groups spanning from 16 to 42[1].

[1] In certain contexts, defining a potential age of treatment makes sense—for instance, in the context of retirement (see Barban et al. 2017).

Table C.2: Example Data Structure - Wide Format I (pers id in column)

		First Birth					Class Sequence				
	age	id 11	id 23	id 44	id 56	id 79	id 11	id 23	id 44	id 56	id 79
↓	20	0	0	0	0	0	A	A	C	A	A
↓	25	0	0	1	0	0	A	A	C	C	A
↓	30	1	0	0	0	0	A	A	A	A	B

Table C.3: Example Data Structure - Wide Format II

	id	Age first birth	20	25	30
1	11	30	A	A	A
2	23	NA	A	A	A
3	44	30	C	C	A
4	56	NA	A	C	A
5	79	NA	A	A	B

C.1.1 Individual Estimates

One solution is to compute an individual estimation for each treated unit. For each treated unit, we pool it with all controls—thus, the controls are potentially used in multiple treated estimations. We refer to this as the “treated pool”.

Within each treated pool, we search for the closest controls. Since the data are drawn from a cohort where all units are observed at the same ages at each period, it is possible to assign a potential treatment timing to each control based on the observed treatment timing in the treated unit.

Table C.4 shows an example of a treated pool. Here, the treatment timing is re-calibrated relative to each treated unit.

By matching each treated pool separately—whether using sequence matching, conventional matching, or a combination thereof—the sequence distance becomes relative to the particular pool. For example, a distance of zero indicates a perfect match within that specific pool.

After matching, we obtain the final dataset, as illustrated in Table C.5, where each treated unit is paired with a matched control.

We can now estimate a matching model using the data from Table C.5, which incorporates both the sequence distance and the set of covariates $\mathbf{X}$.

C.2 Modeling

Our empirical strategy combines traditional covariate adjustment with innovative sequence-based measures that capture the dynamics of life course trajectories. In

Table C.4: Example Data Structure for Individual Estimation (Treated Pool)

unique id	treatment →	0	0	1
	age →	20	25	30
11	treated 1	A	A	A
23	control 1	A	A	A
56	control 2	C	C	A
79	control 3	A	A	B

unique id	treatment →	0	1	0
	age →	20	25	30
44	treated 2	C	C	A
23	control 1	A	A	A
56	control 2	C	C	A
79	control 3	A	A	B

...

Table C.5: Data Structure for Final Matching

id		pair	**X**	seq distance	
11	treated id 1	1	x_i	0	ref treated
23	control id 1	1	x_i	0	control
44	treated id 2	2	x_i	0	ref treated
56	control id 2	2	x_i	0	control

addition to standard confounders (as described in Section B), we integrate three key sequence measures derived from the Optimal Matching (OM) procedure:

- **Sequence Distance** (S_i): Quantifies the dissimilarity between an individual's life course sequence and that of the corresponding matched control (a value of 0 indicates a perfect match).
- **Number of Transitions** (R_i): Counts the number of state changes within a sequence. For example, the sequence `A-A-A-A-B-B-A-D-D-D` involves 3 transitions Gabadinho et al. (2011).
- **Within-Sequence Entropy** (E_i): Measures the Shannon entropy of the sequence, computed as:

$$h(p_1, \ldots, p_s) = -\sum_{i=1}^{s} p_i \log(p_i),$$

where s is the number of distinct states and p_i is the proportion of state i in the sequence (Gabadinho et al., 2011).

These sequence measures capture complex, dynamic information about individuals' pre-treatment trajectories that is often missed by traditional covariates.

C.2.1 Mahalanobis Distance Matching Specification

These sequence-derived measures (S_i, R_i, and E_i) are combined with a set of covariates ($\mathbf{X}_i$) considered as potential confounders. The matching is performed using 1:1 Mahalanobis distance nearest neighbor matching, implemented with the MatchIt package (Stuart et al., 2011).

The Mahalanobis distance between treated ($T_i = 1$) and control ($T_i = 0$) units is calculated using the vector of variables containing:

- The sequence metrics: sequence distance (S_i), number of transitions (R_i), and sequence entropy (E_i)
- Standard confounders ($\mathbf{X}_i$) including:
  - A dummy variable for social origin (indicating professional-class household, i.e., at least one parent in a professional occupation)
  - A dummy variable for maternal employment during childhood
  - A categorical variable for region of birth

The Mahalanobis distance provides a multivariate metric that accounts for the covariance between variables when matching treated and control units, without requiring propensity score estimation. This approach directly matches individuals based on their similarity across all specified covariates and sequence measures.

This combined model yields a propensity score for each individual that reflects both the static and dynamic aspects of their pre-treatment trajectories.

C.2.2 Estimating the Treatment Effect

After matching, each treated unit (mother) is paired with a matched control (childless woman), and we estimate linear probability models (LPMs) on the matched dataset. These models predict the probability that an individual belongs to occupational class c at time t. Time is explicitly incorporated into the model both as a main effect and in interaction with the treatment indicator, allowing us to examine whether the effect of motherhood on occupational outcomes varies over time.

We specify the following regression model:

$$\Pr(Y_{i,t} = c) = \alpha + \delta D_i + \tau t + \gamma(D_i \times t) + \varepsilon_{it}$$

where:

- $Y_{i,t}$ is a binary indicator equal to 1 if individual i belongs to occupational class c at time t, and 0 otherwise
- D_i is the treatment indicator (1 = mother, 0 = childless)
- t represents time periods
- $D_i \times t$ is an interaction term to test whether the effect of motherhood varies over time
- ε_{it} is the error term

C.2.3 Standard Errors

To obtain valid standard errors, we implement robust corrections by clustering at the matched pair level—this adjustment accounts for the reuse of controls (matching with replacement). Additionally, we apply clustering at the individual level to yield conservative standard error estimates. These corrections are implemented using the `vcovCL` function from the `library(sandwich)` in R, following the procedure outlined in Stuart et al. (2011).

Appendix D

Matching Strategy

We use a matching strategy in two stages. First, we retrieve a sequence distance by performing a sequence matching for each treated and its “treated pool” (see Section C.1.1).

We use the Optimal Matching algorithm with substitution cost weight of 2 and indel cost of 1. We use normalised distance (normalised over 2).

Secondly, we use a general matching strategy mixing the sequence distance and the confounders described in Section B. We use a 1:1 Mahalabonis propensity score matching with Nearest Neighbour Matching to estimate the treatment effect using the MatchIt package (Stuart et al., 2011). The advantage of the Mahalabonis matching over the traditional propensity score is discussed in King and Nielsen (2019).

All the analyses are conducted using R (R Core Team, 2013).

D.1 Matching Quality

The strength of causal evidence relies on the question: can controls serve as counterfactuals? Two main criteria are generally considered:

- The balance of pre-treatment confounders (Stuart, 2010)
- The quality of pre-treatment outcome trend (Abadie et al., 2010)

D.1.1 Models

We test 3 main models in according to our theory of potential confounders.

We present one set of models on the full analytical sample and one set of model on the preference sample (see Section A.2.2).

Table D.2, its related Figures D.1 and D.2 shows 4 models for both the **Full-Sample** and the **Sub-Sample Preferences** denoted "Pref" in the models.

(1) The baseline model (called Base), (2) the matching with the OM sequence distance measure (Model A), (3) the matching with the covariates Social Origin, (4) Mother Working at age 16, Region and preferences for the preference sample.

- Baseline Model (Base)
- Model A: Optimal Matching + Number of Transitions + Entropy
- Model B: Social Origin + Mother Working [+ Preferences for sub-sampe]
- Model C: Optimal Matching + Number of Transitions + Entropy + Social Origin + Mother Working [+ Preferences for sub-sample]

Inspecting Table D.2 and Figure D.1 and D.2 suggest the following. While Model A, B, C re-weight the covariates differently, with as expected Model 2 (covariates without the sequence distance), balancing the covariates the best, none of the different matching are fundamentally different when it comes to the estimation of Social Class age Age 42.

Model B s the most at odd with compared to Model A and C regarding Class at age 42. Model B does not account for the sequence distance, and thus is expected to be outperformed by Model A and C. However, Model C (the model incorporating the sequence distance, the sequence entropy and number of transition as well as the confounders/covariates) only slightly differ from the empty Baseline model. It attenuated slightly the proportion of women in Class 1 (-5.5% for the Baseline vs -4% for Model C), and accentuate slighly the proportion for Class 6 (+4.6% vs +6.7%).

All in all, this suggest that selection into motherhood is not strong and is unlikely to yields large biases.

It is as if we could take the observed different between mothers and childless women for the causal effect:

$$\mathbb{E}[Y|D=1] - \mathbb{E}[Y|D=0] \approx \mathbb{E}[Y(1) - Y(0)|D=1] \tag{D.1}$$

While the difference is small between the matching and the raw difference, we will use the matching for the small improvement in balance it yields. We use Model C, the most complete model in our final results.

Conclusion about Preferences

Regarding the importance of preferences, our conclusions suggest that yes preferences matter for later life outcomes, but are not confounders for motherhood and

subsequence outcome trajectories. If this is the case, as table D.3 further suggest, then preferences do not need to be accounted for motherhood penalty models, at least when it comes to social class. Preferences have their own independent effects (i.e. orthogonal). If we take at face value the regressions displayed in Table D.3, strong family and work preferences have their effect "canceled" (in the sense of their size) by education and social origin. In other words, strong family preferences and strong preferences for regular hours at age 16 years old have a negative impact on reaching a Professional social class, while coming from a Professional household and having had a mother working at age 16 years old have a positive effect of reaching a Professional class at age 42.

Table D.1: Covariate Balance Before and After Matching for Full Sample

model	X1	X2	X3	X4	X5	X6	X7	X8	X9	X10	X11	X12	X13	X14	X15
Baseline	-0.049	-0.004				-0.055	-0.089	0.002	-0.007	-0.000	0.046	0.005	0.003	0.127	-0.032
Baseline Pref	-0.023	0.004	-0.157	-0.149	-0.147	-0.054	-0.058	-0.002	-0.007	0.005	0.048	0.002	0.005	0.113	-0.052
Model A	-0.085	-0.025				-0.059	-0.065	-0.014	-0.021	0.004	0.051	-0.007	0.007	0.127	-0.023
Model A Pref	-0.044	-0.008	-0.303	-0.199	-0.141	-0.058	-0.018	-0.018	0.001	-0.008	0.043	0.000	0.005	0.116	-0.064
Model B	-0.002	0.002				0.016	-0.140	0.060	-0.091	0.003	0.061	0.015	0.008	0.128	-0.060
Model B Pref	-0.020	0.017	0.015	-0.045	-0.075	-0.041	0.028	-0.038	0.005	-0.035	0.041	-0.032	0.005	0.108	-0.041
Model C	-0.017	0.011				-0.040	-0.089	-0.015	-0.014	0.004	0.067	-0.005	0.004	0.128	-0.039
Model C Pref	-0.060	-0.005	-0.045	-0.067	-0.154	-0.048	-0.010	-0.050	0.008	-0.036	0.063	-0.013	0.005	0.114	-0.034

Variable labels: A = mother working, B = social origin prof, C = family pref, D = work hours pref, E = reading score
F = Class 1 %, G = Class 2 %, H = Class 3 %, I = Class 4 %, J = Class 5 %, K = Class 6 %, L = Class 7 %
M = Class 8 %, N = Class 9 %, O = Class 10 %
Variable C, D, E are measured at age 16 years old
Social Class is measured at age 42 years old

Table D.2: Covariate Balance Before and After Matching for Preference Sub-Sample

model	A	B	C	D	E	F	G	H	I	J	K	L	M	N	O
Baseline	-0.049	-0.004				-0.055	-0.089	0.002	-0.007	-0.000	0.046	0.005	0.003	0.127	-0.032
Baseline Pref	-0.023	0.004	-0.157	-0.149	-0.147	-0.054	-0.058	-0.002	-0.007	0.005	0.048	0.002	0.005	0.113	-0.052
Model A	-0.085	-0.025				-0.059	-0.065	-0.014	-0.021	0.004	0.051	-0.007	0.007	0.127	-0.023
Model A Pref	-0.044	-0.008	-0.303	-0.199	-0.141	-0.058	-0.018	-0.018	0.001	-0.008	0.043	0.000	0.005	0.116	-0.064
Model B	-0.002	0.002				0.016	-0.140	0.060	-0.091	0.003	0.061	0.015	0.008	0.128	-0.060
Model B Pref	-0.003	0.013	-0.168	-0.229	-0.077	-0.001	-0.028	0.002	-0.091	0.001	0.063	-0.008	0.005	0.100	-0.043
Model C	-0.017	0.011				-0.040	-0.089	-0.015	-0.014	0.004	0.067	-0.005	0.004	0.128	-0.039
Model C Pref	-0.026	0.017	-0.181	-0.140	-0.147	-0.040	-0.023	-0.039	0.001	-0.023	0.063	-0.012	0.005	0.109	-0.041

Variable labels: A = mother working, B = social origin prof, C = family pref, D = work hours pref, E = reading score
F = Class 1 %, G = Class 2 %, H = Class 3 %, I = Class 4 %, J = Class 5 %, K = Class 6 %, L = Class 7 %
M = Class 8 %, N = Class 9 %, O = Class 10 %
Variable C, D, E are measured at age 16 years old
Social Class is measured at age 42 years old

Figure D.1: Covariate Balance Before and After Matching for Full Sample and Preference Sample

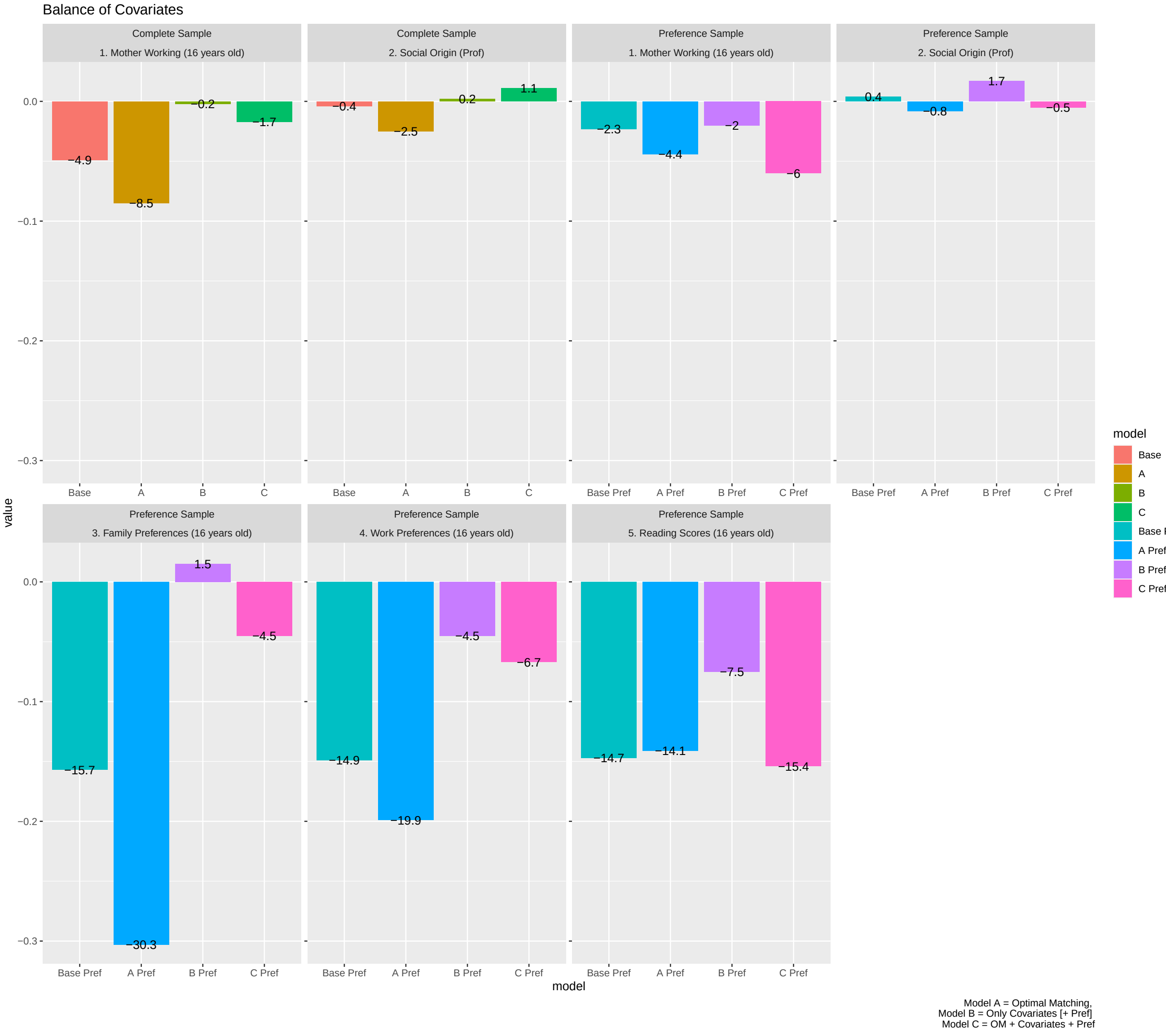

Figure D.2: Covariate Balance Before and After Matching for Full Sample and Preference Sample. Outcomes.

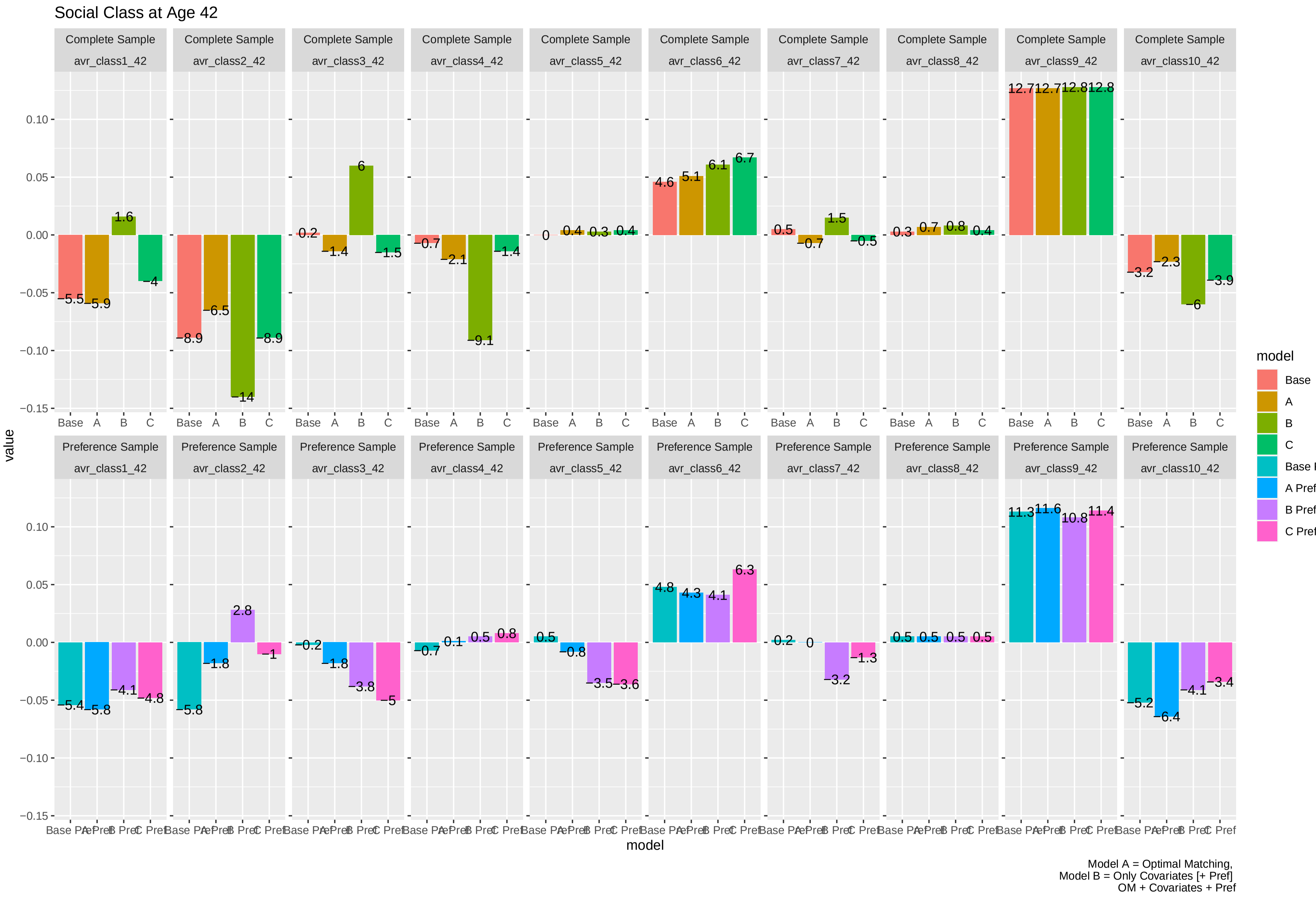

Table D.3: OLS Regression Predicting Social Class at Age 42

	Dependent variable:					
	Social Class: High Prof %			Social Class: Low Prof %		
	(1)	(2)	(3)	(4)	(5)	(6)
Treated (first birth)	−0.055*** (0.015)	−0.053*** (0.015)	−0.043** (0.020)	−0.089*** (0.023)	−0.087*** (0.023)	−0.072** (0.029)
Social origin prof		0.070*** (0.013)	0.062*** (0.018)		0.038* (0.021)	0.045* (0.026)
Mother working at 16		0.035*** (0.013)	0.030* (0.017)		0.035* (0.019)	0.027 (0.025)
Fam pref			−0.025 (0.016)			−0.026 (0.024)
Workhours pref			−0.044** (0.018)			−0.071*** (0.026)
Constant	0.147*** (0.013)	0.106*** (0.015)	0.139*** (0.021)	0.393*** (0.020)	0.363*** (0.023)	0.402*** (0.031)
Observations	2,541	2,541	1,568	2,541	2,541	1,568
R^2	0.005	0.024	0.025	0.006	0.010	0.015

Note: *p<0.1; **p<0.05; ***p<0.01

Family preference = Interested in family life: dummy “1 Very interested”
Work hours preference = Job: To have a job with regular hours: dummy “1 Matters very much”

Appendix E

The Potential Outcomes Framework

E.1 Introduction

Let us describe the potential outcomes framework directly referring to the substantial question of this paper(Morgan and Winship, 2015; Imbens and Rubin, 2015). Let the outcome variable Y denotes women's occupational careers. The potential outcomes framework asks the following question: what would have happened to a women's class trajectory, if she would not have had a child. In this paper, the treatment of interest D is having a child, with $D = 1$ if a women had a child and $D = 0$ if she did not. The potential outcomes framework asks question at the individual-level, hence the subscript i.

δ_i is the individual causal effect.

$$\delta_i = Y_i^{D=1} - Y_i^{D=0} \tag{E.1}$$

Because an individual i is either observed or unobserved (treated or untreated), we can never simultaneously observe Y_i^1 and Y_i^0, because Y_i^1 necessarily implies $D = 1$. We would like to estimate $[Y_i^{D=1}|D_i = 1 - Y_i^{D=0} \mid D_i = 1]$ but $Y_i^{D=0} \mid D_i = 1$ is unobserved.

The estimand of interest is the Average Treatment on the Treated (ATT)

$$E[\delta_i \mid D = 1] = E[Y^1 \mid D = 1] - E[Y^0 \mid D = 1] \tag{E.2}$$

But problematically we only observe

$$E[\delta_i \mid D = 1] = E[Y^1 \mid D = 1] - E[Y^0 \mid D = 0] \tag{E.3}$$

Equation (3) is subject to the following bias

$$E[Y^1 \mid D=1] - E[Y^0 \mid D=0] = E[\delta_i] + E[Y^0 \mid D=1] - E[Y^0 \mid D=0] \quad \text{(E.4)}$$

$E[\delta_i]$ being the true effect and $E[Y^0 \mid D=1] - E[Y^0 \mid D=0]$, the baseline bias or selection bias (Morgan and Winship, p.59). In the absence of treatment Y^0, how do the treated $D=1$ and the controls $D=0$, on average, perform? To estimate correctly the true causal effect, we either need to assume or empirical adjust this difference (or bias) so it equals 0, with $E[Y^0 \mid D=1] - E[Y^0 \mid D=0] = 0$

Various forms of matching or reweighting are used to find appropriate controls (Diamond and Sekhon 2012; Iacus, King, and Porro 2012; Morgan and Harding 2006). Call Z a factor (e.g. stratifying variable) such that once conditioned on, the treatment and outcome become conditionally independent,

$$(Y^0, Y^1) \perp\!\!\!\perp D \mid Z \quad \text{(E.5)}$$

Recently methodologies have proposed to include trajectories or trends in the outcome variable to control for unobservable characteristics (Abadie et al., 2010; Vagni and Breen, 2021) thus reducing bias due to time-varying unobservable characteristics (Xu, 2017). If the ignorability condition holds, then we can use the control group as counterfactual for estimating causal effects.

E.1.1 Average and Individual Effects

Most matching methods focus on average effects and therefore adjust or balance the characteristics of the treated and control cases at an aggregate level. This is so because it is generally accepted that individual-level causal effects cannot be estimated (Morgan and Winship 2007:46). However, the Synthetic Control method has shown that it was possible to retrieve causal estimates at the unit level (Abadie et al., 2015). Vagni and Breen applied what they called the Individual Synthetic Control to individual cases to retrieve individual-level estimates. One of the main benefit of individual estimates is that the study of heterogeneous effect is straightforward. Another benefit is that it enables to study treatment that varies in their timing between treated cases (Vagni and Breen 2021).

We follow the framework of individual effects of Vagni and Breen (2021).

Let w be a counter-factual weight (as calculated by the Synthetic Control Method for instance) used to adjust the treated and control cases. At the individual-level this would simply be

$$Y_i^* = \sum_{j=1}^{J} Y_j^0 w_j \quad \text{(E.6)}$$

Y_i^* then serves as an individual counter-factual estimate, which is a weighted sum of controls.

The individual effect is estimated with

$$\hat{\delta}_i = Y_i^1 - Y_i^* \tag{E.7}$$

Y_i^* is an estimation of $Y_i^0|D = 1$.

The important point is that if the estimation succeed, then the individual baseline bias will be 0 for average individual effect. Any bias results from the difference between Y_i^* and $Y_i^0 \mid D = 1$.

Aggregated into the ATT, which is the average of individual effects, we simply have

$$\begin{aligned} \hat{\delta} &= \frac{1}{N}\sum_{i=1}^{N}\hat{\delta}_i \\ &= \frac{1}{N}\sum_{i=1}^{N}[(Y_i^1|D=1) - (Y_i^0|D=1)^*] \end{aligned} \tag{E.8}$$

Appendix F

BHPS

Our analyses rely on the Understanding Society: Waves 1-8, 2009-2017 and Harmonised BHPS: Waves 1-18, 1991-2009 (Data Archive Study Number 6614).

"British Household Panel Survey (BHPS) and Understanding Society to explore the question of the motherhood class penalty in the UK. The two combined surveys cover the years 1991-2017. We select all women aged 16 to 30 years old at their first observation period. We defined a woman as belonging to the treatment group if she had a child between 1991 and 2017. We only select the treated cases with at least 4 pre-treatment periods of observation and 3 post-treatment observation periods (7 years minimum in total), which excludes mothers aged less than 20 years old."

We have in total 425 treated cases and 1256 control cases (see Online Appendix Table A.2). The average age at first birth is 30 years old. Table A.3. of the Online Appendix shows the number of children by year for treated and control cases. We see that treated by design starts having children in 1995 and this number goes up to a maximum of an average of 1.8 children. Table A.4 and A.5 of the Online Appendix provides further information about the number of cases in each year. The treatment timing (see table A.4 the Online Appendix) variable is constructed in reference to the birth of the first child with 0 indicating the year of the birth. A few numbers of treated cases have been observed for a substantial period of time, with some cases for instance observed first at 17 years old in 1991, who had a child at 36 years old in 2010, and exited the panel at 42 in 2006 (23 years of observation). However, there are unfortunately few of cases like this and it was difficult to find an appropriate number of controls for them, we restricted the window of observations to four years prior to the treatment years and 10 years post-treatment (including the treatment year). Therefore, our analyses are restricted to the effects of the birth of the first child up to 10 years after the birth.

F.1 Sample Selection

The data were prepared as follow.

The birth of the first child is the treatment variable. It was derived from the original variable called `ch1bydv`. We selected all the women with *a least* 4 observations prior to treatment and *a least* 3 observations post-treatments (plus the treatment year) for a total of minimum 8 waves of observations, as illustrated in table F.1

Table F.1: Treatment and Timing Selection

Treatment	0	0	0	0	1	1	1	1
Timing	-4	-3	-2	-1	0	1	2	3

In order to create a meaningful sample for our study, we restricted the age to women aged 16 to 30 years old at the first wave of observation. (This is not necessarily Wave 1 of the panel.) We selected control cases with more than 7 waves of observations. We removed women who ever had a child. The control group is composed of women who never had a child and the treated group is composed of women who had a child during the BHPS or Understanding Society.

Table **??** shows the numbers of treated cases for different pre- and post-treatment observations. We can see that overall there are not many motherhood transitions in the BHPS. Even if we were to subset only women with 1 pre-treatment and 1 post-treatment period, we would only have 747 treated cases.

Table F.2: Descriptive Statistics. N Treated and Controls.

	treated_case	n	avr_age_at_first_birth	avr_age
1	0.00	1256		32.25
2	1.00	425	30.31	33.14

Table F.3: Pre-Treatment Characteristics Comparison between Treated (1) and Controls (0)

	variable	treated	-4	-3	-2	-1	0
1	year	controls	1995.98	1996.98	1997.98	1998.98	1999.98
2	year	treated	1995.98	1996.98	1997.98	1998.98	1999.98
3	age	controls	27.02	28.00	29.01	30.01	31.01
4	age	treated	26.03	27.02	28.05	29.05	30.04
5	earnings	controls	1189.24	1301.64	1418.05	1558.28	1696.02
6	earnings	treated	1643.22	1794.89	1885.45	1912.28	1571.14
7	uni	controls	0.12	0.14	0.16	0.18	0.22
8	uni	treated	0.25	0.26	0.26	0.27	0.28
9	wkhours	controls	23.84	25.62	26.96	28.19	29.29
10	wkhours	treated	31.10	31.61	32.35	31.37	23.81
11	class1	controls	0.04	0.03	0.04	0.04	0.05
12	class1	treated	0.09	0.10	0.11	0.10	0.10
13	class2	controls	0.16	0.18	0.21	0.23	0.26
14	class2	treated	0.27	0.28	0.30	0.31	0.24
15	class3	controls	0.26	0.27	0.28	0.29	0.30
16	class3	treated	0.31	0.31	0.29	0.30	0.24
17	class4	controls	0.24	0.24	0.22	0.19	0.17
18	class4	treated	0.15	0.13	0.13	0.12	0.11
19	class5	controls	0.08	0.07	0.06	0.06	0.06
20	class5	treated	0.04	0.05	0.06	0.05	0.03
21	class6	controls	0.24	0.21	0.20	0.19	0.17
22	class6	treated	0.14	0.13	0.11	0.12	0.28

F.2 Main Results BHPS

Figure F.1: BHPS Average Class Outcomes

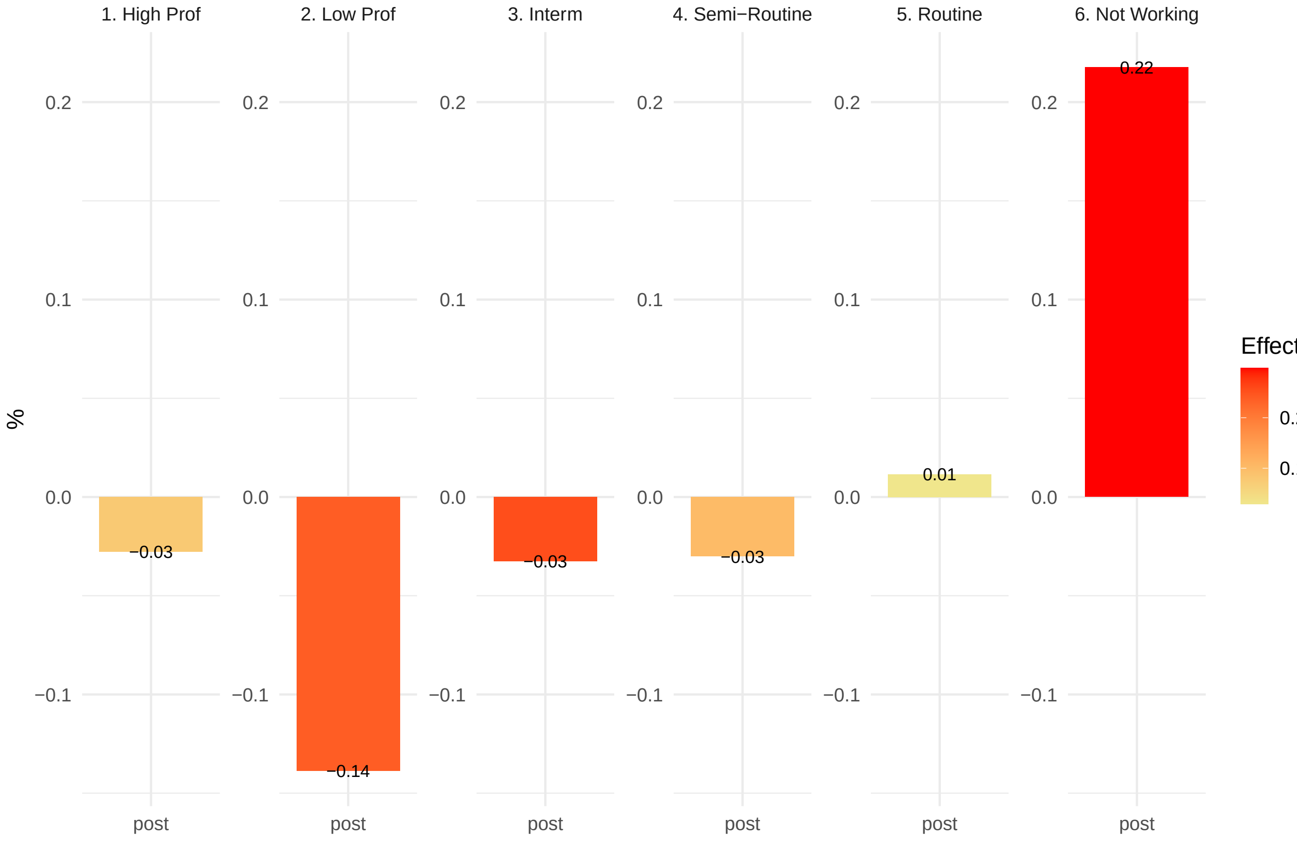

Appendix G

Clusters

G.1 SeqIplot

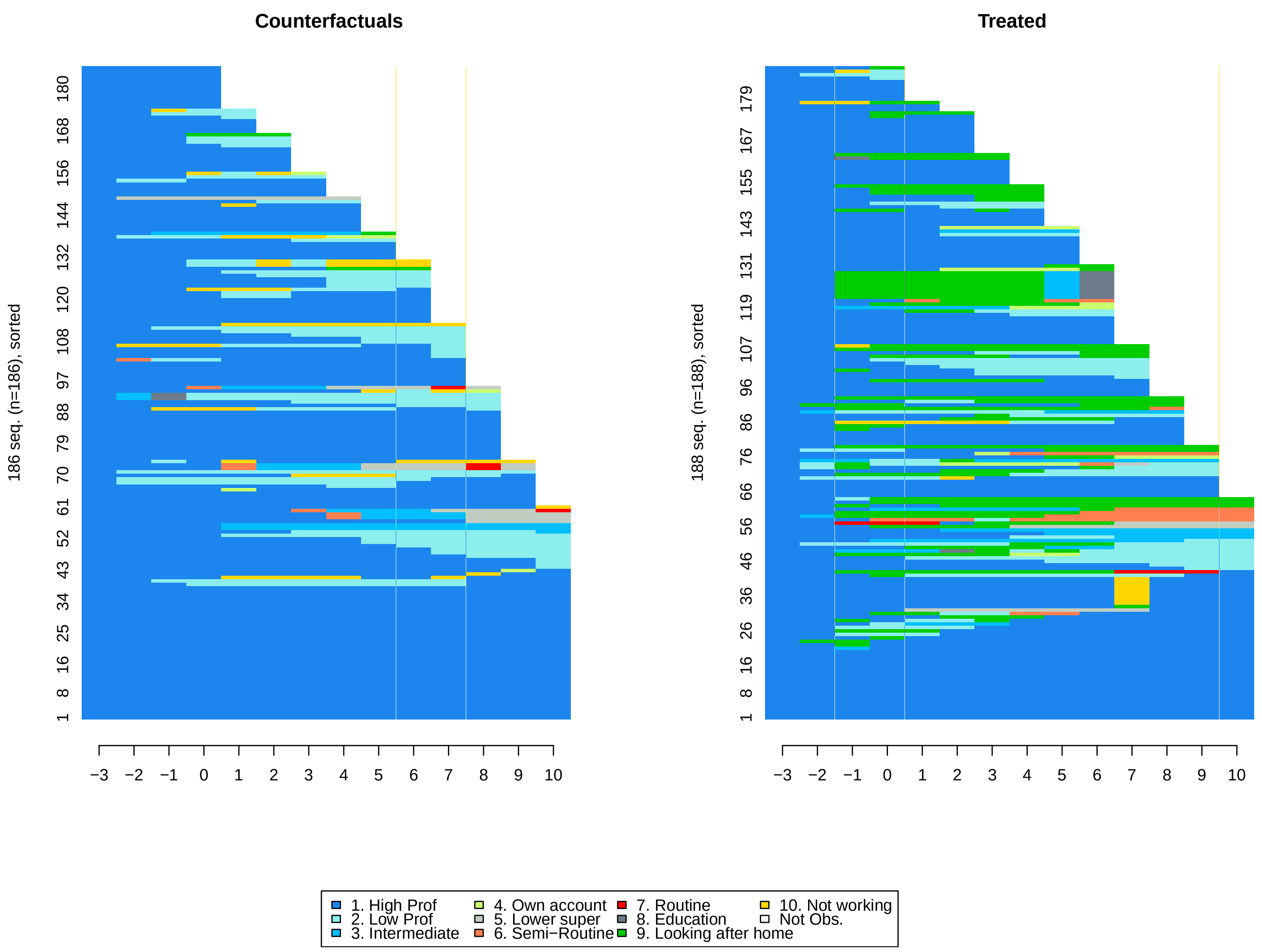

Figure G.1: Sequence Index Plot of Class Trajectories for Women Starting in Class 1. Each line represents an individual trajectory from 3 years before birth (3) to 10 years after birth (0 = year of birth). The vertical axis displays class membership, while the horizontal axis indicates time relative to childbirth. Trajectories are aligned at year 0 to highlight patterns before and after birth.

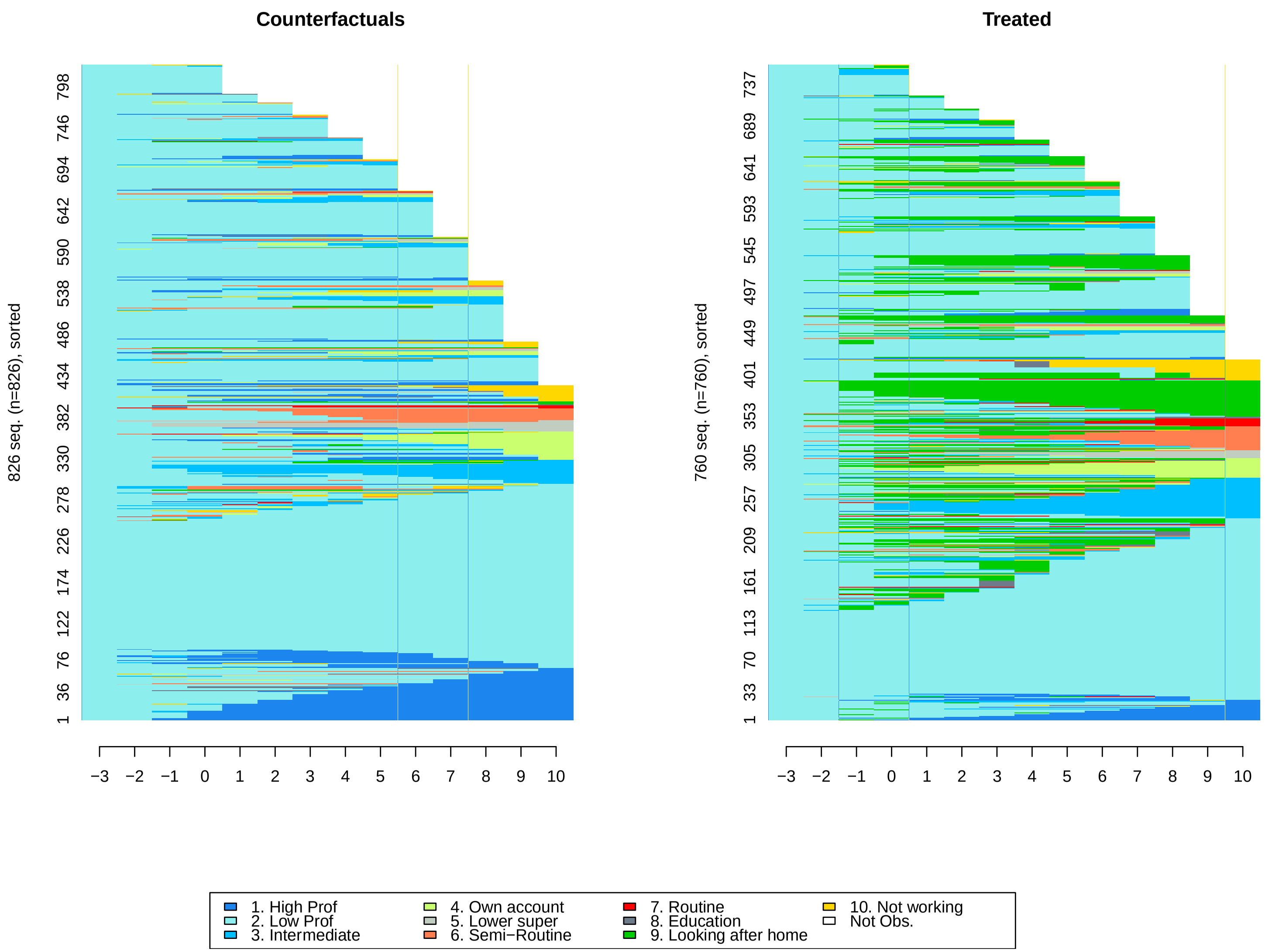

Figure G.2: Sequence Index Plot of Class Trajectories for Women Starting in Class 2. Each line represents an individual trajectory from 3 years before birth (3) to 10 years after birth (0 = year of birth). The vertical axis displays class membership, while the horizontal axis indicates time relative to childbirth. Trajectories are aligned at year 0 to highlight patterns before and after birth.

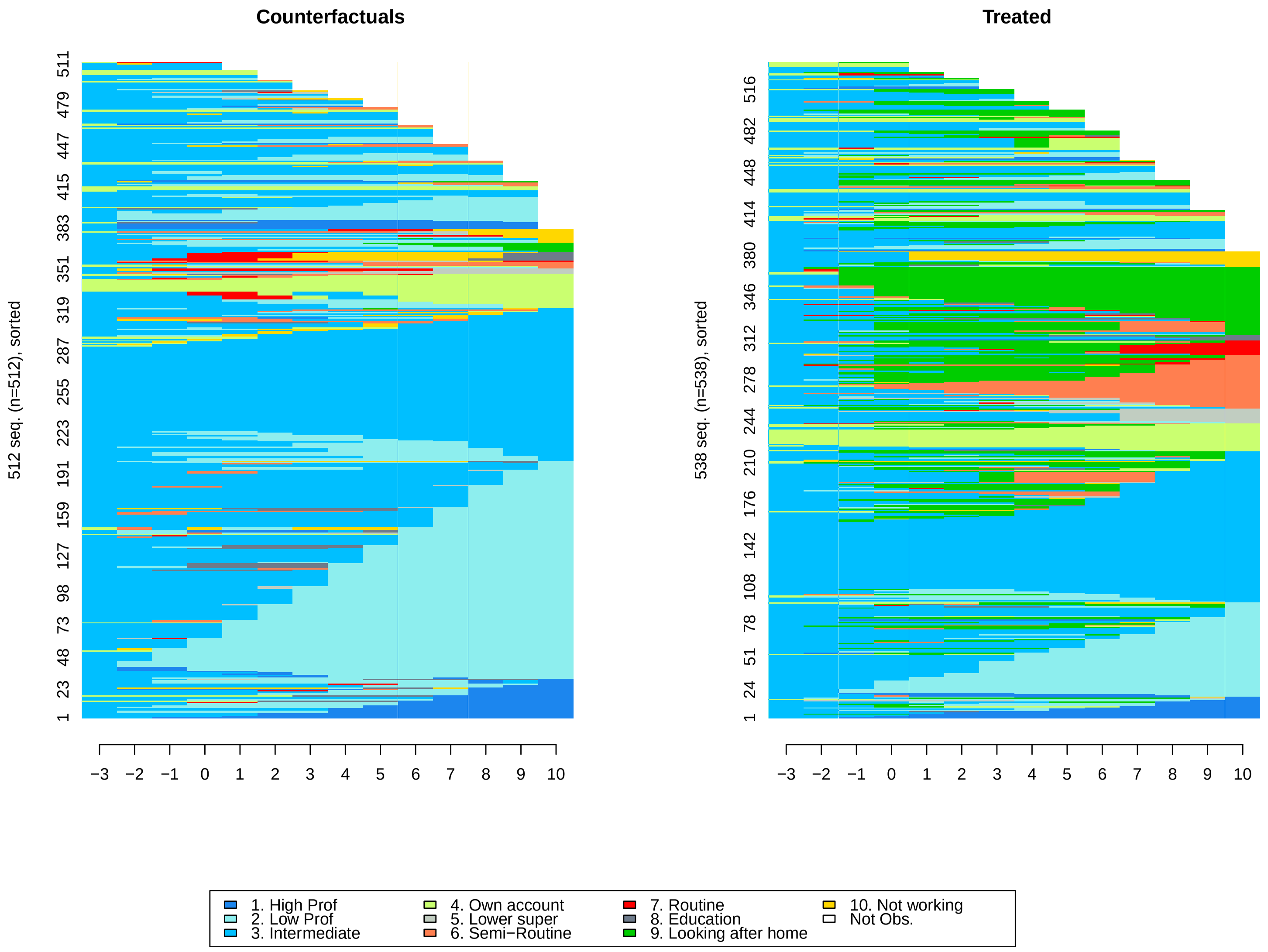

Figure G.3: Sequence Index Plot of Class Trajectories for Women Starting in Class 3. Each line represents an individual trajectory from 3 years before birth (3) to 10 years after birth (0 = year of birth). The vertical axis displays class membership, while the horizontal axis indicates time relative to childbirth. Trajectories are aligned at year 0 to highlight patterns before and after birth.

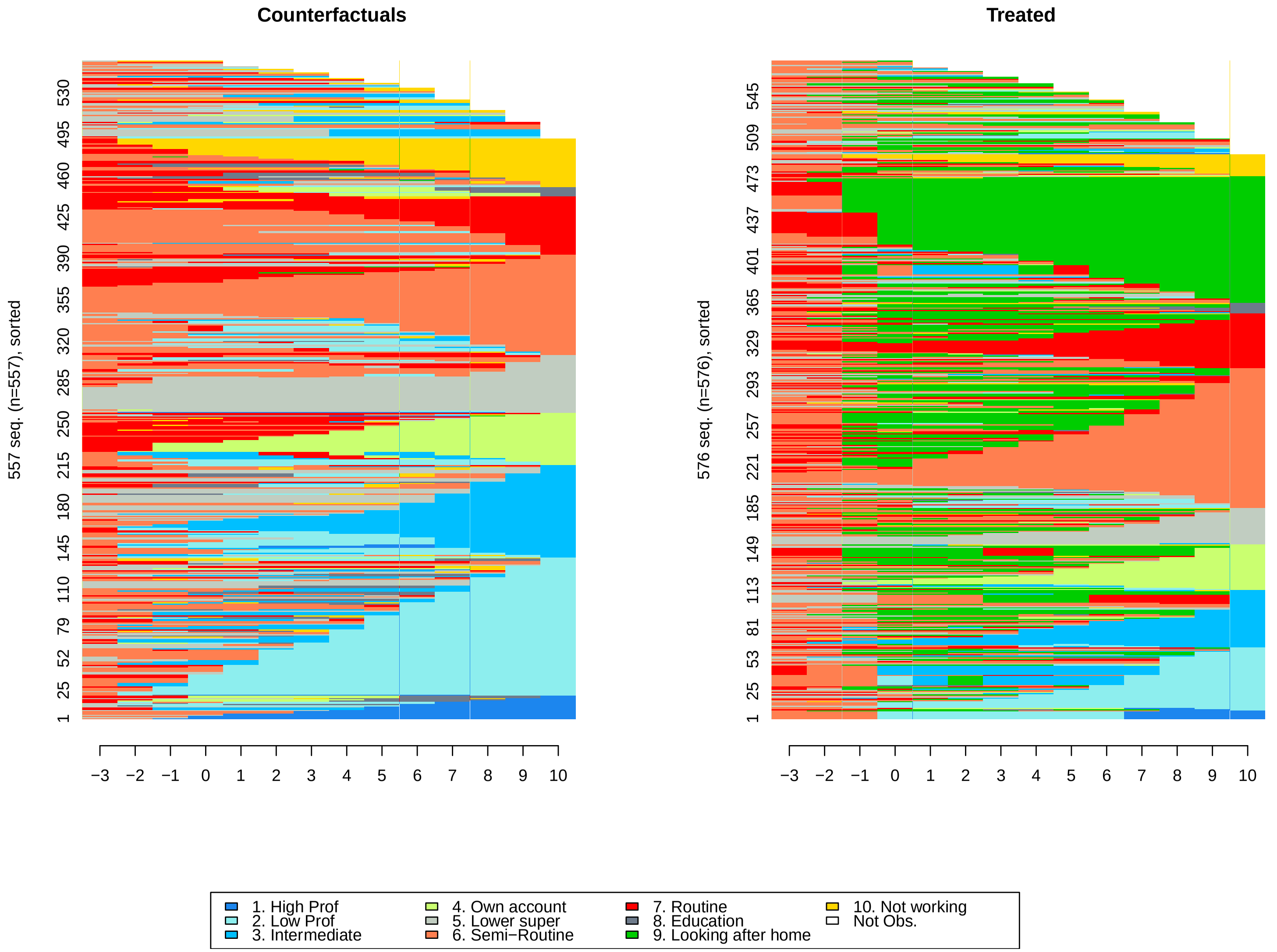

Figure G.4: Sequence Index Plot of Class Trajectories for Women Starting in Class 6. Each line represents an individual trajectory from 3 years before birth (3) to 10 years after birth (0 = year of birth). The vertical axis displays class membership, while the horizontal axis indicates time relative to childbirth. Trajectories are aligned at year 0 to highlight patterns before and after birth.

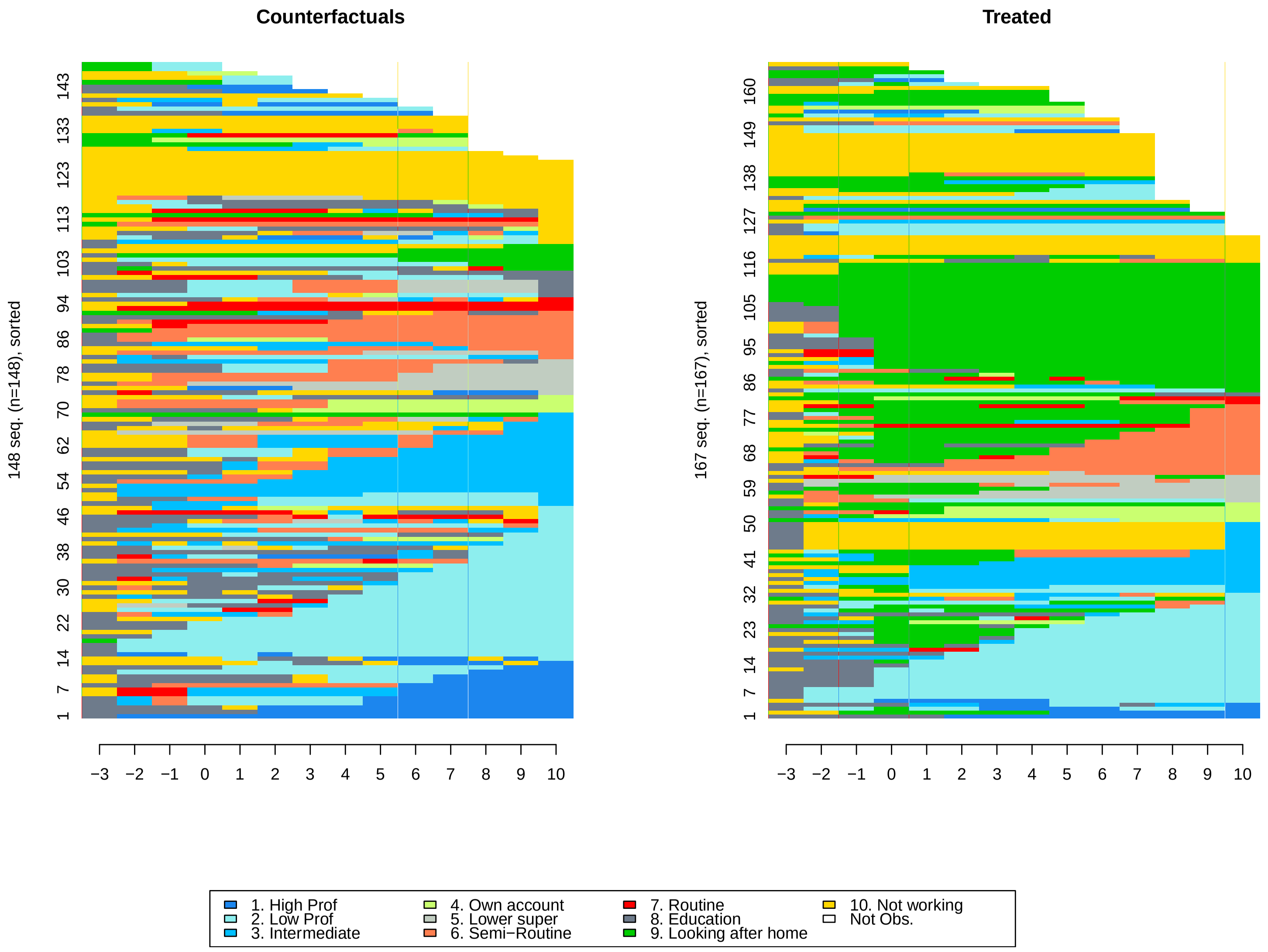

Figure G.5: Sequence Index Plot of Class Trajectories for Women Starting in Class 8-9-10. Each line represents an individual trajectory from 3 years before birth (3) to 10 years after birth (0 = year of birth). The vertical axis displays class membership, while the horizontal axis indicates time relative to childbirth. Trajectories are aligned at year 0 to highlight patterns before and after birth.

G.2 Cluster Working Class

We explore in more detail the heterogeneity in women's Working Class trajectory by running a cluster analysis on their class sequence. We took all the women in a working class occupation 3 years before birth (those we see in Figure 4 in the main manuscript).

Four main clusters summarise the trajectories.

The proportion of the 4 clusters are as follow: 39.2%, 27%, 16.6% and 17.2%.

We can see a great deal of upward mobility in 3 of the 4 clusters.

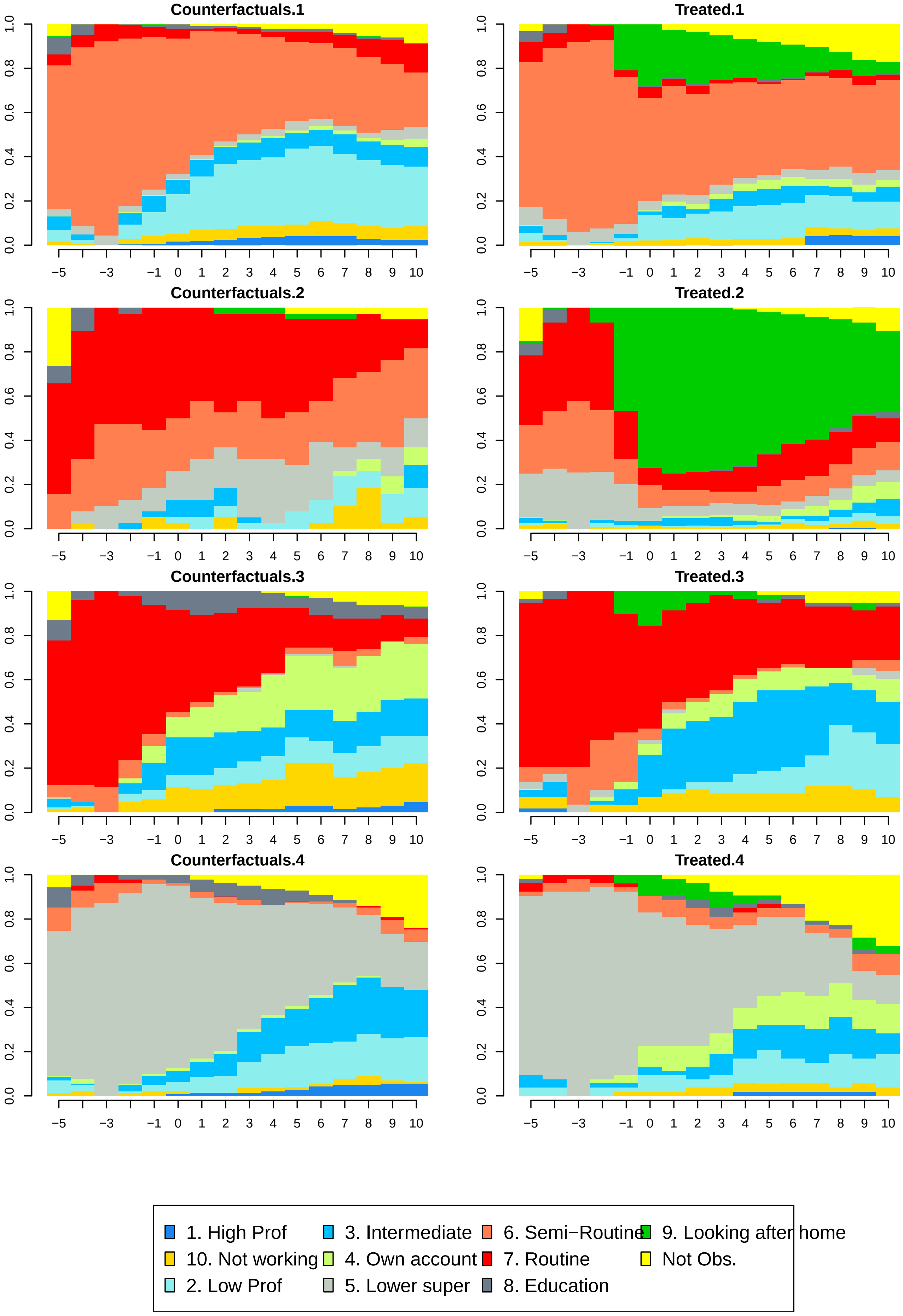

Figure G.6: Pre-Treatment Working Class Clustering

References

Abadie, A., Diamond, A., and Hainmueller, J. (2010). Synthetic control methods for comparative case studies: Estimating the effect of california's tobacco control program. *Journal of the American statistical Association*, 105(490):493–505.

Abadie, A., Diamond, A., and Hainmueller, J. (2015). Comparative politics and the synthetic control method. *American Journal of Political Science*, 59(2):495–510.

Gabadinho, A., Ritschard, G., Müller, N. S., and Studer, M. (2011). Analyzing and visualizing state sequences in r with traminer. *Journal of Statistical Software*, 40(4):1–37.

Imbens, G. W. and Rubin, D. B. (2015). *Causal inference in statistics, social, and biomedical sciences*. Cambridge University Press.

King, G. and Nielsen, R. (2019). Why propensity scores should not be used for matching. *Political Analysis*, 27(4):435–454.

Morgan, S. L. and Winship, C. (2015). *Counterfactuals and causal inference*. Cambridge University Press.

R Core Team (2013). *R: A Language and Environment for Statistical Computing*. R Foundation for Statistical Computing, Vienna, Austria.

Stuart, E. A. (2010). Matching methods for causal inference: A review and a look forward. *Statistical science: a review journal of the Institute of Mathematical Statistics*, 25(1):1.

Stuart, E. A., King, G., Imai, K., and Ho, D. (2011). Matchit: nonparametric preprocessing for parametric causal inference. *Journal of statistical software*.

Vagni, G. and Breen, R. (2021). Earnings and income penalties for motherhood: estimates for british women using the individual synthetic control method. *European Sociological Review*, 37(5):834–848.

Xu, Y. (2017). Generalized synthetic control method: Causal inference with interactive fixed effects models. *Political Analysis*, 25(1):57–76.
